# Dynamic regulation of Pin1 expression and function during zebrafish development

**DOI:** 10.1371/journal.pone.0175939

**Published:** 2017-04-20

**Authors:** Maria Solange Ibarra, Carla Borini Etichetti, Carolina Di Benedetto, Germán L. Rosano, Ezequiel Margarit, Giannino Del Sal, Marina Mione, Javier Girardini

**Affiliations:** 1 Instituto de Biología Molecular y Celular de Rosario (IBR), Consejo Nacional de Investigaciones Científicas y Técnicas (CONICET), Rosario, Argentina; 2 Centro de Estudios Fotosintéticos y Bioquímicos (CEFOBI), Consejo Nacional de Investigaciones Científicas y Técnicas (CONICET), Rosario, Argentina; 3 Laboratorio Nazionale CIB, Area Science Park Padriciano, Trieste, Italy; 4 Dipartimento di Scienze della Vita-Università degli Studi di Trieste, Trieste, Italy; 5 Centre for Integrative Biology—CIBIO, University of Trento, Trento, Italy; Washington State University, UNITED STATES

## Abstract

The prolyl isomerase Pin1 plays a key role in the modulation of proline-directed phosphorylation signaling by inducing local conformational changes in phosphorylated protein substrates. Extensive studies showed different roles for Pin1 in physiological processes and pathological conditions such as cancer and neurodegenerative diseases. However, there are still several unanswered questions regarding its biological role. Notably, despite evidences from cultured cells showing that Pin1 expression and activity may be regulated by different mechanisms, little is known on their relevance *in vivo*. Using *Danio rerio* (zebrafish) as a vertebrate model organism we showed that *pin1* expression is regulated during embryogenesis to achieve specific mRNA and protein distribution patterns. Moreover, we found different subcellular distribution in particular stages and cell types and we extended the study of Pin1 expression to the adult zebrafish brain. The analysis of Pin1 overexpression showed alterations on zebrafish development and the presence of p53-dependent apoptosis. Collectively, our results suggest that specific mechanisms are operated in different cell types to regulate Pin1 function.

## Introduction

Signal transduction mechanisms make use of phosphorylation reactions to achieve rapid and reversible regulation of pathways underlying cell behavior. The enzymes that catalyze phosphorylation reactions are classified in Ser/Thr or Tyr kinases, according to substrate specificity. Among Ser/Thr kinases, those that preferentially phosphorylate Ser or Thr residues preceding Pro in protein substrates (S/T-P motifs) are known as proline-directed kinases. This group includes several kinases from the Cyclin-dependent kinase (CDKs), Extracellular signal–Regulated kinase (ERKs), p38, and Jun N-terminal kinase (JNKs) families, as well as Glycogen synthase kinase 3 (GSK3), Polo-Like Kinase 1 (PLK1) and Mechanistic Target of Rapamycin (mTOR) among others [1]. A unique feature of signaling pathways that include proline-directed phosphorylation is that they may be further regulated by post-phosphorylation conformational changes through isomerization of the peptide bond preceding Pro [2]. Due to the large free energy difference between the *cis* and *trans* conformations, peptide bonds are allowed to adopt only the *trans* conformation. However, the presence of the five-membered ring and the imide in peptide bonds preceding Pro lowers this difference allowing both conformations. Notwithstanding, the spontaneous conversion is extremely slow. Pin1 catalyzes the *cis-trans* isomerization of the peptidyl-prolyl bond in S/T-P motifs, taking the process to the nano second scale, which is useful for the rapid events required in signal transduction. Upon isomerization, local conformational changes are induced on protein substrates that lead to changes in activity, stability, subcellular localization or susceptibility to receive other post-translational modifications [3]. In this way, Pin1 transduces S/T-P phosphorylation events into functional changes in protein substrates.

Human Pin1 is a monomeric enzyme of 163 amino acids consisting of two domains [4]. The WW domain on the N-terminus binds specifically to phosphorylated S/T-P motifs [5]. The C-terminus contains the peptidyl-prolyl isomerase (PPIase) domain, which is responsible for the catalytic activity [6]. Several proteins involved in a wide variety of cellular processes were identified as Pin1 substrates (reviewed in [3]). Following the initial evidences suggesting that Pin1 may negatively regulate mitosis entry, a growing body of data has proposed a more extended role as part of checkpoint mechanisms in the cell cycle. For example, Pin1 prevents mitotic entry by inducing hBora degradation and inhibiting CDC25 catalytic activity [7]. However, after mitotic entry, Pin1 may cooperate with M phase progression through its action on Aurora A [8] and WEE [9]. In contrast, the role of Pin1 on G1/S transition is less clear. While the described effects of Pin1 on CyclinD1 [10] and Retinoblastoma protein (pRb) [11] favor the transition to S phase, its action on p53[12], p27 [13] and cyclin E [14] promotes cell cycle arrest. Nevertheless, it is now clear that Pin1 is involved in other cellular processes. A role for Pin1 in mRNA biosynthesis and processing was proposed following the discovery that Pin1 regulates eukaryotic RNA polymerase II [15]. Likewise, Pin1 was shown to interact with proteins that regulate mRNA decay such as KH-type splicing regulatory protein (KSRP), Human antigen R protein (HuR) and AU-rich element RNA-binding protein 1 (AUF1) [16, 17]. In addition, Pin1 was shown to interact with cell signaling and cytoskeletal proteins [3]. Pin1 was also involved in stress responses most likely as a modulator of the concerted action of p53 family members [18]. In response to stress activated kinases, Pin1 promotes p53 stabilization and enhances its ability to organize active transcriptional complexes on target promoters, thereby promoting both nuclear and cytoplasmic p53 pro-apoptotic activities [12, 19–21]. Pin1 was also shown to promote TAp63 [22] and TAp73 [23] stabilization and apoptotic function, as well as to bind ΔN isoforms [23, 24].

Besides being involved in physiological processes, a large body of evidences has shown that Pin1 may affect pathological conditions. Pin1 function was shown to be inhibited in humans with Alzheimer´s disease [25]. Moreover, Pin1 overexpression in postnatal neurons *in vivo* protects against neurodegeneration in a mouse model for Alzheimer´s disease [26]. However, in other neurodegenerative conditions Pin1 seems to cooperate with pathological mechanisms. In a mouse model of Huntington’s disease Pin1 promoted p53 activation and cooperated with mutant Huntingtin to engage p53-dependent apoptosis [27]. Likewise, Pin1 was shown to promote the formation of α-synuclein inclusions, and elevated protein levels were observed in human *postmortem* Parkinson’s Disease brains [28].

A complex picture was also depicted on the role of Pin1 in cancer. Clinical studies have shown that Pin1 is frequently overexpressed in different cancers [29] and in some cases its overexpression correlates with clinical outcome [30–32]. Moreover, *in vitro* studies have shown the ability of Pin1 to foster oncogenic mechanisms and *in vivo* models of breast cancer support the notion that Pin1 overexpression may favor tumor development [33–35]. However, the ability to induce degradation of pro-oncogenic proteins such as c-Myc and cyclin-E suggests that, depending on the combination of genetic and epigenetic alterations underlying the pathology, Pin1 may cooperate with tumor suppression as well. Indeed, c-Myc mutants unable to bind Pin1 showed an increased tumorigenic potential [36], and Pin1 knock-out mouse embryonic fibroblasts (MEFs) were more prone to transformation induced by RAS GV12 and p53 inhibition [14]. Moreover, Pin1 was shown to reduce proliferation *in vitro* and tumor growth in a xenograft model using renal cell carcinoma cell lines [37].

Collectively, the experimental evidences suggest that Pin1 acts as a global regulator of phosphorylation signaling, through the integration of different pathways. However, the wide range of identified and potential substrates, as well as the dependence on phosphorylation for Pin1 binding, has complicated the rationalization of its biological role. The involvement of Pin1 in such different processes suggests that its expression and activity should be under tight control. In most normal human tissues Pin1 shows a moderate expression, with elevated levels in particular cell types [29]. Pin1 expression was also shown to be comparable between mouse organs including brain, testis, lung, liver and mammary epithelium [38]. However, the correlation between Pin1 overexpression and cancer progression suggests that stringent control of Pin1 levels is important for normal physiology. Pin1 transcription is activated by E2 factor (E2F) [39] and Notch Intracellular Domain (N1ICD) [40] and repressed by the Activating Enhancer Binding Protein 4 (AP4) [41]. In addition, Pin1 was shown to be ubiquitylated and degraded by the proteasome [42]. However, little is known on the involvement of the aforementioned mechanisms in determining Pin1 levels *in vivo*. Although less studied, evidences on Pin1 phosphorylation suggest a complex regulatory network, since both activating and inhibitory modifications were reported. Several kinases were shown to phosphorylate S16 in the WW domain, including Protein kinase A (PKA) [43] and Aurora A [7]. This modification was associated with inhibition of binding ability and nuclear localization. Similarly, Death-associated protein kinase 1 (DAPK1) inhibits catalytic activity and nuclear localization by phosphorylating S71 [44]. On the contrary, Mixed Lineage Kinase 3 (MLK3) phosphorylates Pin1 on S138 increasing its activity and nuclear translocation [45]. In addition, reduced ubiquitylation and degradation were observed upon phosphorylation on S65 by PLK1 [42].

The characterization of Pin1 function and regulation is of fundamental importance to understand the mechanisms that allow precise signal transduction and integration. Evidences from cell lines have provided valuable knowledge on Pin1 effects on specific substrates. However, considering the dependence of Pin1 activity on cell type and context, studies in animal models may contribute to understand more deeply its biological role. In order to gain insights into Pin1 function *in vivo* we characterized its expression during embryogenesis using zebrafish as a model organism, and we studied the consequences of Pin1 overexpression. We have also analyzed the presence of Pin1 in the adult zebrafish brain. This strategy allowed us to unveil previously unappreciated aspects of the regulation of Pin1 expression and activity.

## Results

### Analysis of *pin1* expression during zebrafish embryonic development

Although several orthologues of human Pin1 have been described previously (reviewed in [46]), the expression and function of Pin1 in zebrafish are still poorly understood. Multiple alignment analysis showed a high similarity between zebrafish Pin1 protein sequence and those from other species reaching almost 80% identity with *Xenopus laevis*, but also with mammals, including *Homo sapiens* (S1A Fig). This high similarity is suggestive of a conserved function for Pin1 in vertebrates. The maximum variability was found in the short flexible region connecting the WW domain with the rotamase domain (amino acids 38–50 in human Pin1). In contrast, residues that were identified as essential for binding to S/T-P motifs and catalytic activity are conserved in all the sequences analyzed. Among them, we identified residues corresponding to W11 and W34 in the human sequence, which are essential to the structure of the WW domain, as well as S16, R17 and Y23, involved in phosphoserine recognition. In addition, K63 and R69, which are essential for binding the phosphate group in the protein substrate, and residues that form the hydrophobic Pro-binding pocket, like L122, M130, Q131 and F134, are also conserved [47].

**Fig 1:**
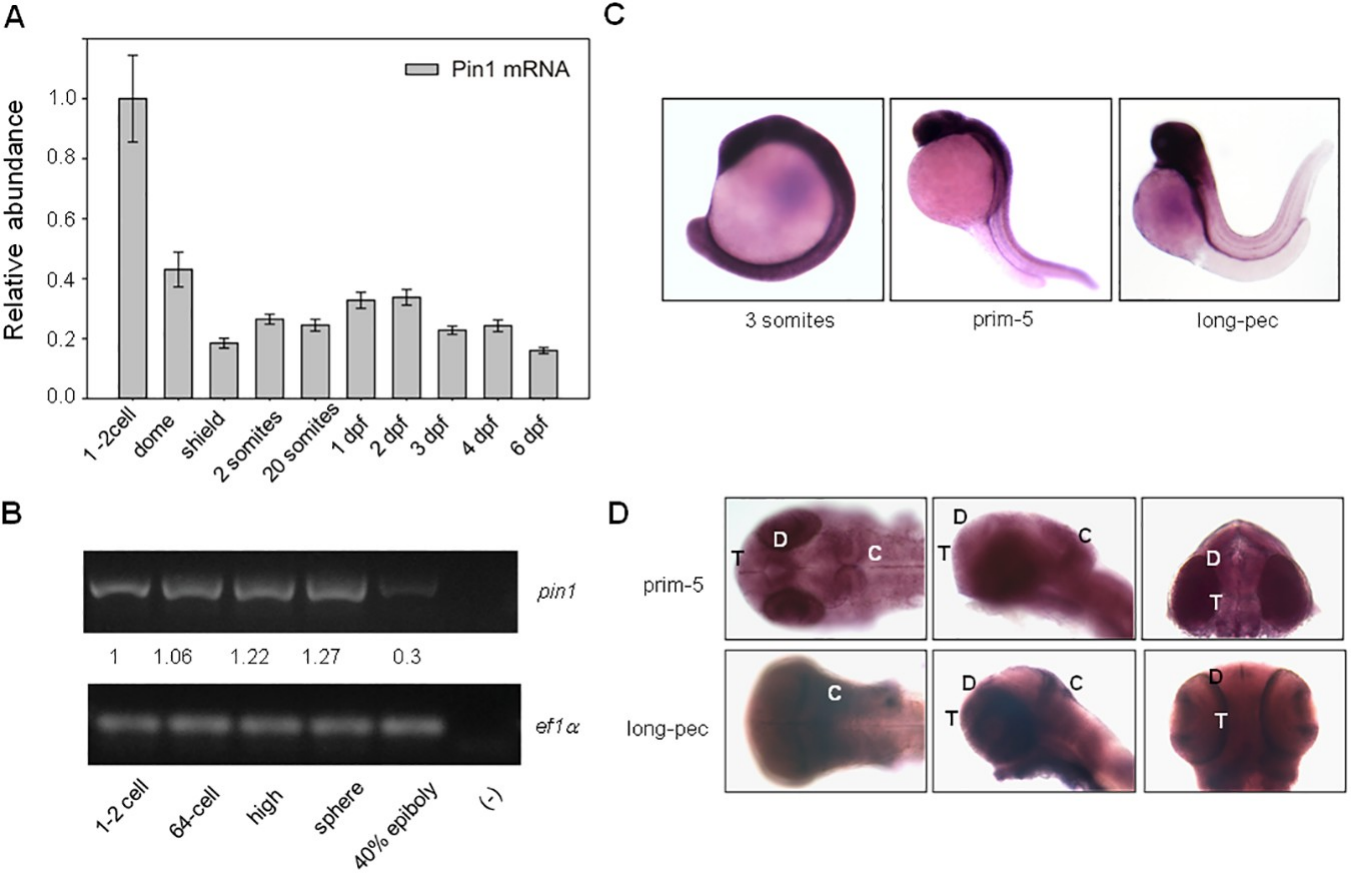
Analysis of *pin1* mRNA levels and distribution during zebrafish development. (A) *pin1* mRNA levels were monitored by RT-qPCR on cDNA from embryos of the indicated stages: 1–2 cell (0 hpf), dome (4:30 hpf), shield (6 hpf), 2 somites (10:30 hpf), 20 somites (19 hpf),1 dpf, 2 dpf, 3 dpf, 4 dpf and 6 dpf. Data was normalized using elongation factor 1*α*(*ef1α*) and ribosomal protein L13a (*rpl13a*) mRNA levels as internal controls and is shown as relative abundance comparing with 1–2 cell stage. (B) Analysis of *pin1* mRNA levels by semi quantitative RT-PCR: 1-2-cell (0 hpf), 64-cell (2 hpf), high (3:30 hpf), sphere (4 hpf) and 40%-epiboly (5 hpf). As an internal control, *ef1α*was amplified on the same cDNA samples. Signal intensity was quantified using ImageJ software and expressed relative to 1–2 cell stage. (C) *pin1* mRNA distribution in whole embryos at the indicated developmental stages analysed by in situ hybridization (100 x magnification). (D) *pin1* mRNA pattern in the head at prim-5 (24 hpf) and long-pec (48 hpf) stages. Left: dorsal, center: lateral, right: frontal (200 x magnification). hpf, hours post-fecundation, dpf, days post-fecundation. T: telencephalon, D: diencephalon, C: cerebellum.

In order to characterize *pin1* expression during development in zebrafish we performed RT-qPCR. Total RNA was isolated from several stages and cDNA was obtained by reverse transcription using oligodT as a primer. Our results showed that *pin1* mRNA is already present at the 1–2 cell stage, indicating that it is maternally inherited. At later stages *pin1* mRNA levels were markedly reduced (Fig 1A). We confirmed these observations by performing semi quantitative RT-PCR at some of the early stages using different oligonucleotides as primers (Fig 1B). These results indicate that *pin1* mRNA levels are subjected to pronounced changes during early development and suggest that a high mRNA concentration may be needed in the egg and/or in developmental stages before *de novo* transcription is activated during the Mid Blastula Transition.

To understand if *pin1* is differentially expressed in specific regions of the embryo, whole mount *in situ* hybridization (WISH) experiments were performed. A specific probe was generated using zebrafish Pin1 coding sequence. To this end, the sequence was amplified by PCR on cDNA from 1–2 cell stage embryos. The DNA fragment was cloned into pGEM-T Easy vector to generate pGPin1 plasmid, which was used as a template for *in vitro* transcription. Digoxigenin-labeled antisense RNA obtained from this reaction was used as a Pin1 probe for WISH, while sense Pin1 RNA was used as a negative control. In three somite (3S) embryos a higher concentration of *pin1* mRNA in the head and anterior part of the trunk was observed (Fig 1C). Notably, a differential expression pattern was even more evident at 24 hours post-fecundation (hpf) and 48 hpf, indicating a striking difference in mRNA levels between the head and the rest of the embryo (Fig 1C). A more detailed analysis of WISH experiments revealed that in 24 hpf embryos *pin1* mRNA was concentrated in the cerebellum, the ventricular zone of the diencephalon and in the thelencephalon (Fig 1D). A similar expression pattern was maintained in 48 hpf embryos where a more intense signal suggests that *pin1* mRNA concentration is increased in the head but remained extremely low in the trunk and tail (Fig 1C). These results show that *pin1* expression is regulated by mechanisms that result in accumulation of mRNA in the embryo brain.

**Fig 2:**
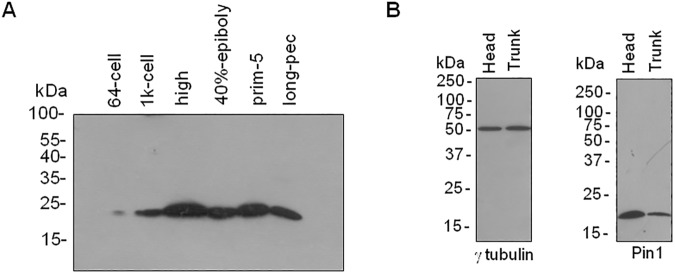
Analysis of Pin1 protein levels in zebrafish embryonic development. (A) Western blot analysis of extracts from 64-cell (2 hpf), 1k-cell (3 hpf), high (3:30 hpf), 40%-epiboly (5 hpf), prim-5 (24 hpf) and long pec (48 hpf) stages using Pin1 antibody. In each case, 40 μg total protein per well were loaded. (B) Western blot analysis of extracts from 48 hpf embryo heads or embryo trunks as indicated, using γ-tubulin antibody as loading control (left panel) or Pin1 antibody (right panel).

In order to analyze Pin1 protein expression we performed western blot analysis on deyolked embryo extracts from different stages using a polyclonal antibody against human Pin1 [19,34]. The ability of the antibody to recognize the zebrafish protein was demonstrated by western blot experiments on lysates from HEK-293 cells that were transfected with pT2Pin1, expressing zebrafish Pin1 fused to EGFP under the control of *ef1α* promoter from *X*. *laevis*. The antibody was able to recognize a band corresponding to zebrafish Pin1-EGFP fusion (~ 43 kDa) only in cells transfected with pT2Pin1, but not with pT2 (S2A Fig). Moreover, endogenous human Pin1 (~18 kDa) from HEK-293 cells was detected in all cases. The presence of the fusion protein was further confirmed probing the membranes with an antibody recognizing GFP (S2A Fig). Western blot analysis on zebrafish extracts allowed us to identify a unique band corresponding to endogenous Pin1 (Fig 2). To further assess the specificity of the antibody we performed pre-absorption assays (S2B Fig). The Pin1 antibody was pre-incubated with purified recombinant human Pin1 fused to GST (GST-hPin1) or with GST as a control. We found that incubation with GST had no effect on the ability of the antibody to recognize the band corresponding to Pin1 on extracts from 4:30 hpf or 24 hpf zebrafish embryos analyzed by western blot (S2B Fig). In contrast, incubation with GST-hPin1 completely blocked the ability of the antibody to recognize the protein. Moreover, we did not observe other bands on our immunoblots. Collectively our results demonstrate that the antibody specifically recognizes zebrafish Pin1.

We then obtained protein extracts from embryos at other stages and the same amount of total proteins of each sample was loaded for western blot analysis. We observed that Pin1 was already present in 64-cell stage (2 hpf) embryos but protein levels were significantly increased at high stage (3:30 hpf), when Mid Blastula Transition is already started in embryos incubated at 28°C (Fig 2A). At later stages, Pin1 protein levels were comparable. Collectively, our results suggest that upon reactivation of *de novo* transcription in the developing embryos, changes on the mechanisms that regulate *pin1* expression are operated to reduce total Pin1 mRNA levels while maintaining protein accumulation. We then asked if Pin1 protein may be present exclusively in the anterior part of the embryo, as suggested by WISH experiments. To answer this question, we sectioned 48 hpf embryos in order to separate the head from the trunk and obtained protein extracts from each sample. Surprisingly, following western blot analysis we found that Pin1 protein was present in both parts of the embryo at comparable levels (Fig 2B). Although normalization with gamma tubulin as a housekeeping suggested that pin1 levels were moderately increased in the head, the protein was readily detectable, in contrast to mRNA levels which were almost absent in the trunk upon 24 hpf.

### Analysis of Pin1 distribution and subcellular localization in zebrafish embryos

To further characterize the presence of Pin1 protein we performed immunofluorescence studies on whole embryos at different stages. Embryos were fixed, probed with anti Pin1 as primary antibody and analyzed by confocal microscopy. As a control for the specificity of the signal, we performed immunofluorescence using the Pin1 antibody pre-incubated with GST-hPin1. As expected, the fluorescent signal was absent when the blocked antibody was used, but not when it was pre-incubated with GST (S2C Fig). To rule out the presence of cross reactions of the Pin1 antibody with unspecific epitopes we also performed immunofluorescence upon *pin1* knock-down. Embryos were injected with morpholinos against zebrafish Pin1 [48] at 1–2 cell stage and analyzed upon 24 hpf. Western blot experiments confirmed that Pin1 protein levels were reduced (S2D Fig). As expected, a marked decrease in Pin1 signal was also observed in whole mount immunofluorescence when embryos were injected with Pin1 morpholinos, comparing with controls (S2E Fig). Therefore, our results show that the signal observed in immunofluorescence studies is specific for zebrafish Pin1.

**Fig 3:**
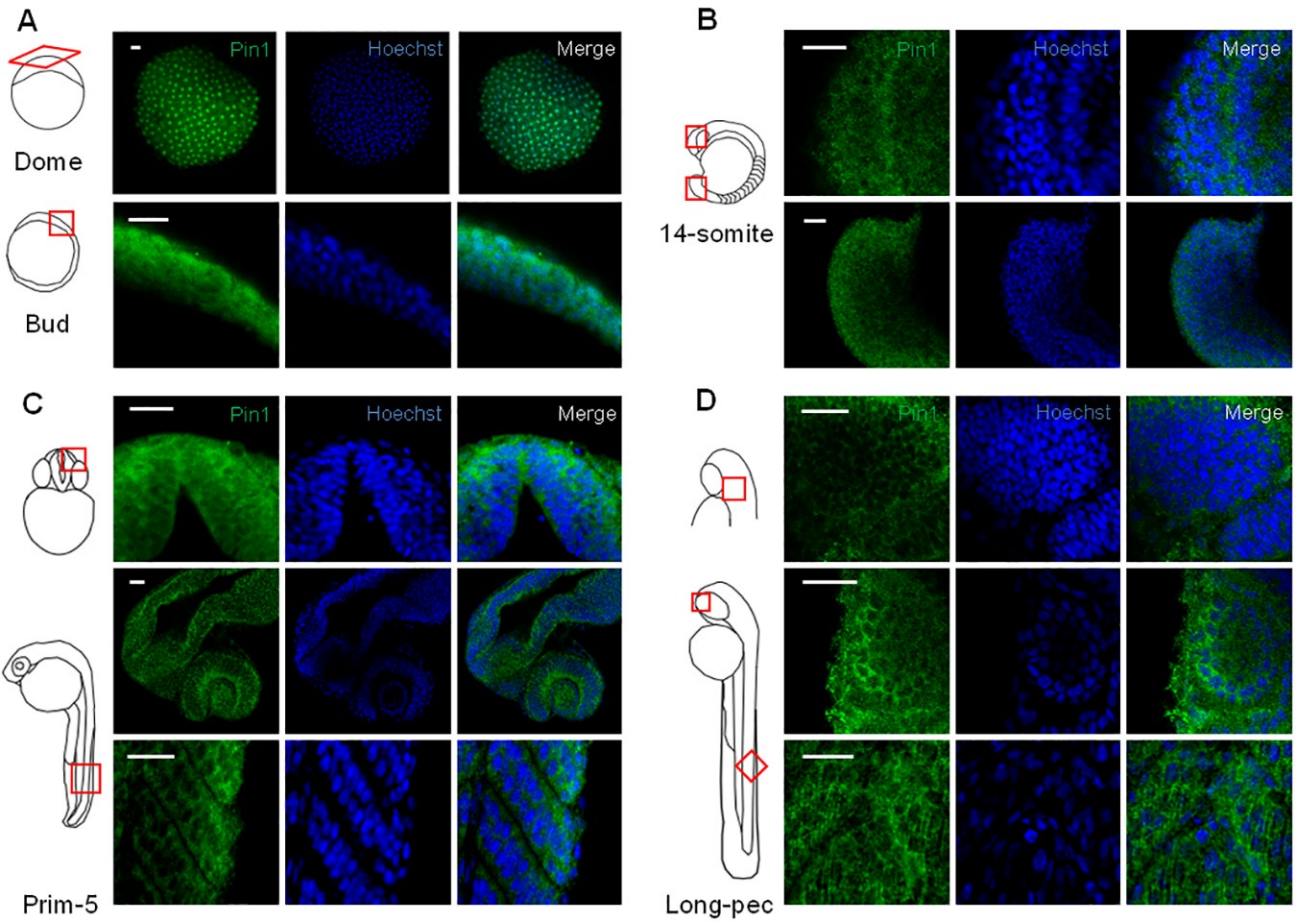
Analysis of Pin1 distribution in zebrafish embryos. Confocal images of whole-mount immunofluorescence performed on embryos from different stages using Pin1 antibody. (A) Dome (4:30 hpf) and Bud stage (10 hpf). (B) 14-somite stage (16 hpf), lateral views from the head (upper panels) and tail (lower panels). (C) Prim-5 stage (24 hpf), frontal view of the head (upper panels), dorsolateral view of the head (middle panels) and lateral view of the tail. (D) Long-pec stage (48 hpf), dorsolateral view of the head (upper panels), lateral view of the head (middle panels) and lateral view of the tail (lower panels). Nuclei were stained with Hoechst. Scale bar = 25 μm.

In agreement with the results from western blot experiments, Pin1 was already present in dome stage (4:30 hpf), where it seems to be concentrated in the nuclei of several analyzed cells (Fig 3A). Analysis of later stages showed that Pin1 expression was maintained in bud stage embryos (10 hpf) but the signal was increased in the cytoplasm. At early somitogenesis (16 hpf), the cytoplasmic Pin1 signal continued to increase in the analyzed areas, while nuclear signal was reduced, suggestive of cytoplasmic accumulation (Fig 3B). This localization was even more clearly observed in the regions analyzed at prim 5 (24 hpf) and long pec stages (48 hpf) including telencephalon, diencephalon, eyes and cerebellum, as well as in myotomes (Fig 3C and 3D). In most cells the nuclear signal was markedly reduced comparing with the cytoplasmic, as judged by co-staining of nuclear DNA with Hoechst, indicating a preferential cytoplasmic localization. In agreement with our results from western blot experiments, we did not find a significant difference in Pin1 staining between the head and trunk of 24 or 48 hpf embryos. Collectively, our results suggest that Pin1 localization changes from predominantly nuclear to cytoplasmic as embryonic development proceeds through the analyzed stages.

We also performed immunofluorescence studies on sections of 48 hpf embryos that were fixed and embedded in paraffin, in order to confirm our observations (S3 Fig). Pin1 presence was found in several regions including telencephalon, diencephalon, eyes, optic tectum and cerebellum. A stronger signal was observed in the cytoplasm of most cells comparing with the nucleus, further supporting the notion that Pin1 was preferentially localized in the cytoplasm. Even if Pin1 subcellular localization was proposed to be dynamically regulated by phosphorylation, most data in the literature reported nuclear accumulation. In order to compare our results from zebrafish embryos with other experimental systems we performed immunofluorescence studies on cultured cells. In agreement with similar reports we observed that Pin1 accumulated in the nucleus of the cell lines analyzed, including neuroblastoma cells (SH-SY5Y, Neuro-2a), both in proliferation conditions and when differentiation was induced by treatment with all-*trans* retinoic acid (S4A Fig). In conclusion, our results indicate that *pin1* expression is regulated by specific mechanisms in different regions of the embryo. Our results also suggest that subcellular localization is highly dynamic, changing from nuclear to cytoplasmic accumulation in different cell contexts.

**Fig 4:**
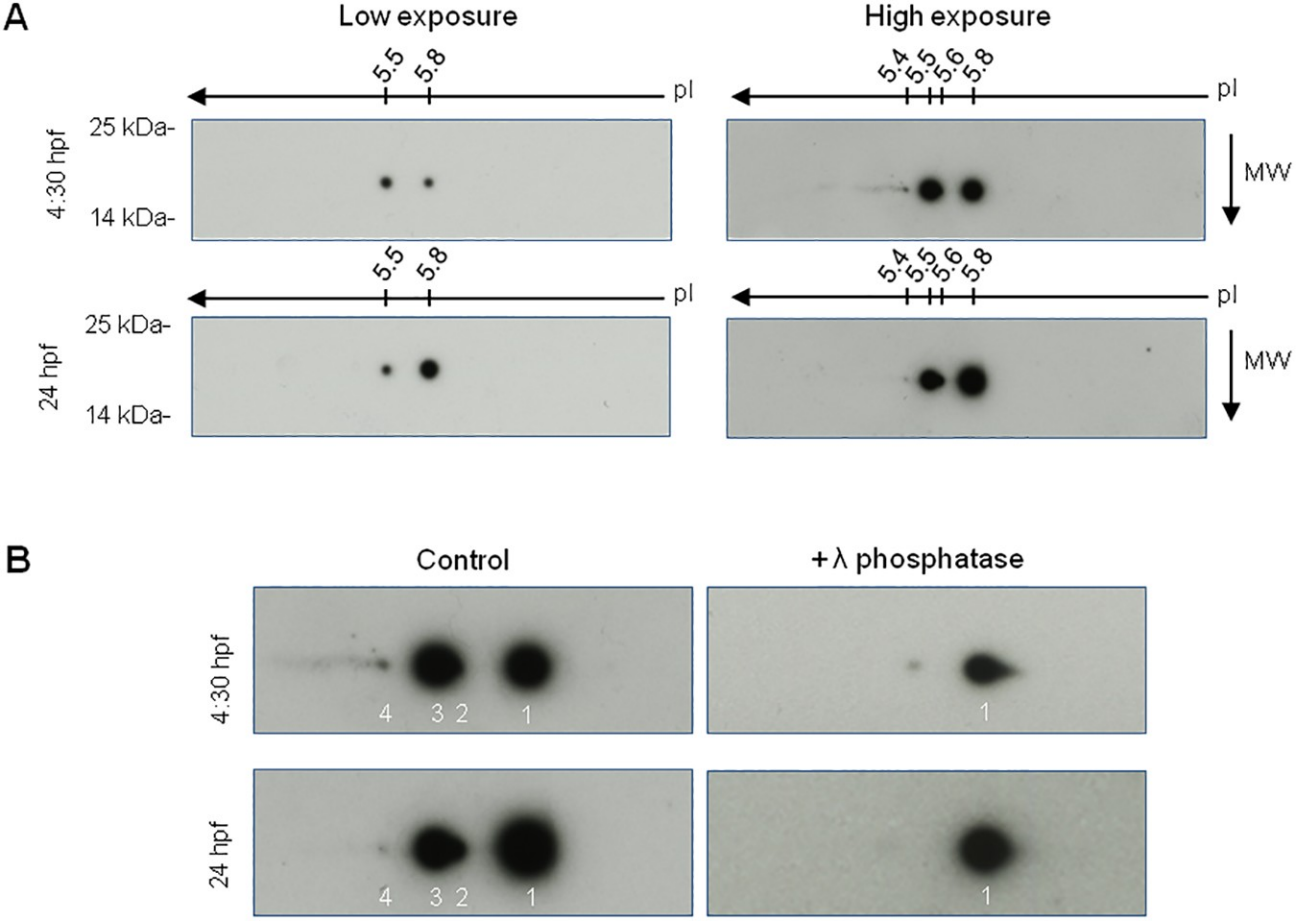
Analysis of Pin1 isovariants in zebrafish extracts. (A) Representative images of two-dimensional (2D) western blot analysis of zebrafish extracts from 4:30 and 24 hpf embryos probed with anti-Pin1. Left and right panels exhibit different exposure times of the same immunoblot. (B) Left panels: magnification of the immunoblots shown in (A). Pin1 isovariants are numbered from the most basic (1) to the most acidic (4). Right panels: 2D western blot analysis of zebrafish extracts from 4:30 and 24 hpf embryos treated with λ phosphatase. pI: isoelectric point, MW: molecular weight.

In order to explore if post-translational modifications may affect Pin1 subcellular localization, we performed bidimensional (2D) denaturant gel electrophoresis followed by western blot at different stages. Extracts from 4:30 hpf and 24 hpf embryos were run and probed with Pin1 antibody. We identified four spots running at 18 kDa in each extract, corresponding to Pin1 isovariants (Fig 4). The most basic form (isovariant 1) showed an isoelectric point of 5.8, in close accordance to the predicted value from Pin1 primary sequence [49]. The remaining spots were resolved at more acidic pHs, indicating the presence of modifications that increase the negative charge of the protein, as for example phosphorylation or acetylation. Interestingly, the relative intensity of the spots was markedly different comparing both stages, indicating changes in the abundance of each modified form. At 4:30 hpf, isovariant 3 was the most abundant and isovariant 4 was clearly detected, even if it was far less abundant respective to 1 and 3. In contrast, isovariant 1 was the most abundant at 24 hpf while the signal corresponding to isovariant 4 was almost negligible. To further characterize the isovariants we performed similar 2D denaturant gel electrophoresis upon treatment of protein extracts with λ phosphatase. A dramatic reduction on the intensity of the spots 2–4 was observed comparing with isovarant 1, demonstrating that the acidic isovariants are phosphorylated forms (Fig 4B). These results strongly suggest that Pin1 phosphorylation is dynamically regulated during development and subscribes the idea that changes on those modifications may contribute to regulate subcellular localization.

### Analysis of Pin1 overexpression in zebrafish embryonic development

**Fig 5:**
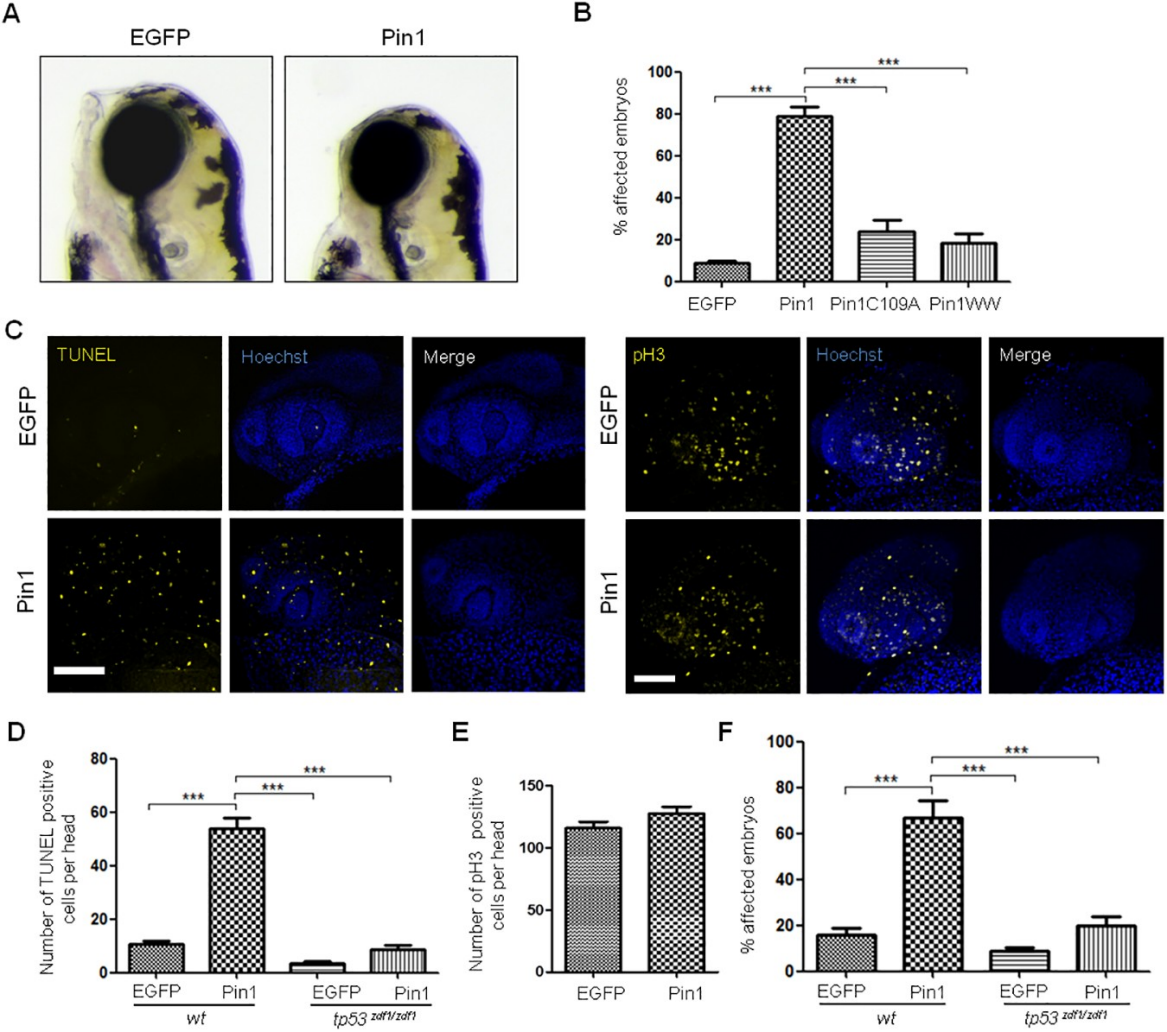
*pin1* mRNA injection affects head development in zebrafish. (A) Lateral views of live 3 dpf embryos injected with EGFP mRNA or EGFP-Pin1 mRNA (Pin1) at 1–2 cell stage. (B) Quantification of the percentage of embryos with altered head development upon injection of mRNAs coding for EGFP, EGFP-Pin1, EGFP-Pin1C109A or EGFP-WW, as indicated. Embryos with evident facial retraction, reduced mandible and/or reduced head size were scored as positive. The graph shows the average of 4 experiments with at least 50 embryos each and the standard deviation. Statistical analysis were carried out using one-way ANOVA followed by Tukey's Multiple Comparison Test: *** p˂ 0,0001. (C) Representative confocal projections of embryos microinjected with EGFP mRNA or EGFP-Pin1 mRNA, stained for apoptosis by whole-mount TUNEL assay (left panels) or for proliferation by p-H3 whole-mount immunofluorescence (right panels). (D) Quantification of apoptotic cells on heads of wild type (wt) or *tp53* ^*zdf1/zdf1*^ embryos microinjected with EGFP mRNA or EGFP-Pin1 mRNA as indicated. Statistical analyses were carried out using one-way ANOVA followed by Tukey's Multiple Comparison Test: *** p˂ 0,0001 (n = 17 for each condition). (E) Quantification of cells positive for p-H3 staining on heads of wild type embryos microinjected with EGFP mRNA or EGFP-Pin1 mRNA as indicated. Statistical analyses were carried out using unpaired t test with Welch's correction (n = 21 for each condition). (F) Morphological analysis at 3 dpf of wild type or *tp53* ^*zdf1/zdf1*^ embryos microinjected with EGFP mRNA or EGFP-Pin1 mRNA as indicated. The graph shows the average of 4 experiments with at least 50 embryos each and the standard deviation. Statistical analysis were carried out using one-way ANOVA followed by Tukey's Multiple Comparison Test: *** p˂ 0,0001.

Next, we decided to explore the role of Pin1 during zebrafish embryogenesis. It has been described that Pin1 downregulation in zebrafish embryos using different morpholino sequences showed negligible effects in the absence of stress situations [48,50]. Given that high Pin1 levels were associated with human pathological conditions, we reasoned that the analysis of its overexpression in zebrafish embryos may provide relevant information on the consequences of Pin deregulation. In order to perform mRNA injection experiments, pCMVSP6-EGFP-Pin1 was constructed. This plasmid contains the zebrafish Pin1 coding sequence fused to EGFP, flanked by the SP6 promoter at the 5’ end and the SV40 polyadenylation signal at the 3’ end. To check the expression of the fusion protein, the plasmid was transfected into HEK-293 cells and the presence of the corresponding band was confirmed by western blot (S4B Fig). As a control, a similar plasmid was generated to synthesize EGFP mRNA. Zebrafish embryos were injected at 1–2 cell stage with EGFP-Pin1 or EGFP mRNA. Embryos showing green fluorescence were selected 5 hours post-injection and incubated at 28°C. Morphological analysis showed that a significant percentage of embryos injected with EGFP-Pin1 mRNA displayed alterations in head development. The alterations were more evident at 3 days post fecundation (dpf), were 79% of embryos showed a smaller mandible, facial retraction or both (Fig 5A and 5B). In several cases, a marked reduction of the frontal telencephalon and head size was observed. In contrast, less than 10% of embryos injected with EGFP mRNA showed some facial alteration or smaller head size. To understand if the observed effects depended on the isomerase activity we microinjected mRNAs containing the sequence of a Pin1 deletion, lacking the catalytic domain but including the WW domain fused to EGFP. In addition, an mRNA coding for Pin1C109A mutant was also injected. This residue corresponds to human C113, which was previously shown to be critical for Pin1 activity. Human Pin1C113A mutant showed more than 90% reduction in isomerase activity, but maintained the ability to bind phosphorylated S/T-P sites [51]. C109 is placed in a stretch of 14 residues that are completely conserved between mammals, zebrafish and *X*. *laevis* (S1A Fig), suggesting that its effect on protein function may be conserved. The expression of both fusion proteins was also confirmed by western blot upon transfection of the corresponding plasmids in HEK-293 cells (S4B Fig). We found that the percentage of 3 dpf embryos with alterations in head development was significantly reduced in embryos injected with EGFP-WW or EGFP-Pin1C109A mRNAs comparing with embryos overexpressing EGFP-Pin1, supporting the notion that isomerase activity is involved in the observed effect (Fig 5B).

Morphological analysis of embryos injected with EGFP-Pin1 mRNA suggested the presence of cell death at prim-5 stage (24 hpf). In order to gain more insights on the effects of Pin1 overexpression we studied the presence of apoptosis using the TUNEL assay (Fig 5C). When we quantified the presence of apoptosis in the head we confirmed that embryos injected with EGFP-Pin1 mRNA showed a higher number of positive cells comparing with controls injected with GFP mRNA (Fig 5D). In contrast, we did not find differences in the number of mitotic cells analyzed by whole mount immunofluorescence with p-H3 antibody between 24 hpf embryos injected with both mRNAs, indicating that Pin1 overexpression did not affect proliferation (Fig 5C and 5E). We performed similar experiments using the zebrafish line *tp53*^*zdf1/zdf1*^, carrying a missense mutation in *tp53* at codon 214, which abrogates p53 apoptotic response [52]. The results obtained showed a remarkable reduction in the number of apoptotic cells upon EGFP-Pin1 mRNA microinjection (Fig 5D), implying that the enhancement of apoptosis upon Pin1 overexpression in wild type embryos depended on active p53. Taken together, our results show that Pin1 overexpression enhances p53-dependent apoptosis at 24 hpf. When we analyzed the morphology of *tp53*^*zdf1/zdf1*^ embryos injected with EGFP-Pin1 mRNA we found a significant decrease in the percentage of embryos displaying alterations in the head (Fig 5F). These results indicate that the effect observed at 3 dpf in wild type embryos depends, at least in part, on p53 and suggest that they are related to apoptosis induced by Pin1 overexpression.

### Pin1 expression in the adult brain

**Fig 6:**
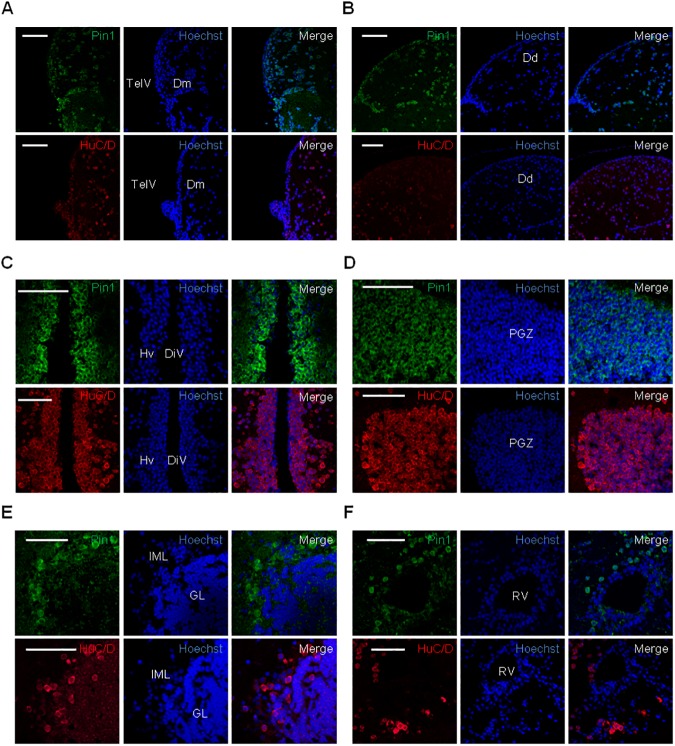
Analysis of Pin1 expression in the adult zebrafish brain. Immunofluorescence analysis on brain coronal sections using Pin1 (green) or HuC/D (red) as primary antibodies. (A) and (B) dorsal telencephalon, (C) ventral diencephalon, (D) optic tectum, (E) corpus cerebelli, (F) romboencephalic ventricular zone. DiV: diencephalic ventricle, Dd: lateral zone of dorsal telencephalon, Dm: medial zone of dorsal telencephalon, GL: cerebellar granular layer, Hv: ventral zone of periventricular hypothalamus, IML: cerebellar intermediate layer, PGZ: periventricular gray zone of the optic tectum, RV: romboencephalic ventricle, TelV: telencephalic ventricle. Scale bar = 50 μm.

Following our results from embryos we wondered if Pin1 expression and subcellular localization may change in different cell types in adult tissues. Some reports have highlighted the involvement of Pin1 in the development and functions of the central nervous system [50,53]. Therefore, in order to expand our observations we decided to study the expression and subcellular localization of Pin1 in the adult brain. We performed immunofluorescence studies on coronal sections of adult zebrafish brains. Serial sectioning of paraffin embedded brains was performed in order to obtain representative samples of different areas from the forebrain to the hindbrain. Sections were subjected to immunofluorescence using anti-Pin1 and analyzed by confocal microscopy. In order to compare with neuron distribution, consecutive sections were probed with HuC/D antibody. In contrast to embryos, were Pin1 protein distribution was more homogeneous, we found Pin1 expression restricted to distinct areas of the regions analyzed (S5 Fig), suggesting that protein expression is differentially regulated in particular cell types. Noteworthy, cells completely negative for Pin1 staining were visible in several regions (S5 and S6 Figs). Extensive areas of Pin1 positive cells were found in the forebrain and midbrain. Pin1 positive cells were scattered throughout the olfactory bulbs. In the telencephalon, Pin1 signal was concentrated in regions adjacent to the lobe surface. A higher concentration of positive cells was found in the inner ventricular surface, but Pin1 expressing cells were also found along the dorsal and lateral surface of the telencephalic lobes (Fig 6A and 6B). In addition, positive cells were observed disseminated in the parenchyma, mostly in the dorsal telencephalic area (S5B Fig). Regions of high accumulation of Pin1 expressing cells were also found close to the diencephalic ventricle (S5C Fig) including the ventral zone of the periventricular hypothalamus (Fig 6C), the central posterior thalamic nucleus and the periventricular nucleus of posterior tuberculum (S6A Fig). In the midbrain, we found extended regions of strong Pin1 signal in the periventricular grey zone of the optic tectum (Fig 6D). In the hindbrain, a strong Pin1 signal was observed in cells dispersed in the central and ventral regions of the medullae oblongata and spinalis (S5 and S6 Figs), including areas near the romboencephalic ventricle (Fig 6F). Dorsally, Pin1 expressing cells were found surrounding the corpus cerebelli, probably as part of the Purkinje layer in the cerebellar intermediate layer (Fig 6E), and caudally, at the border of the lobus vagus and near the lobus facialis (S6E and S6F Fig).

**Fig 7:**
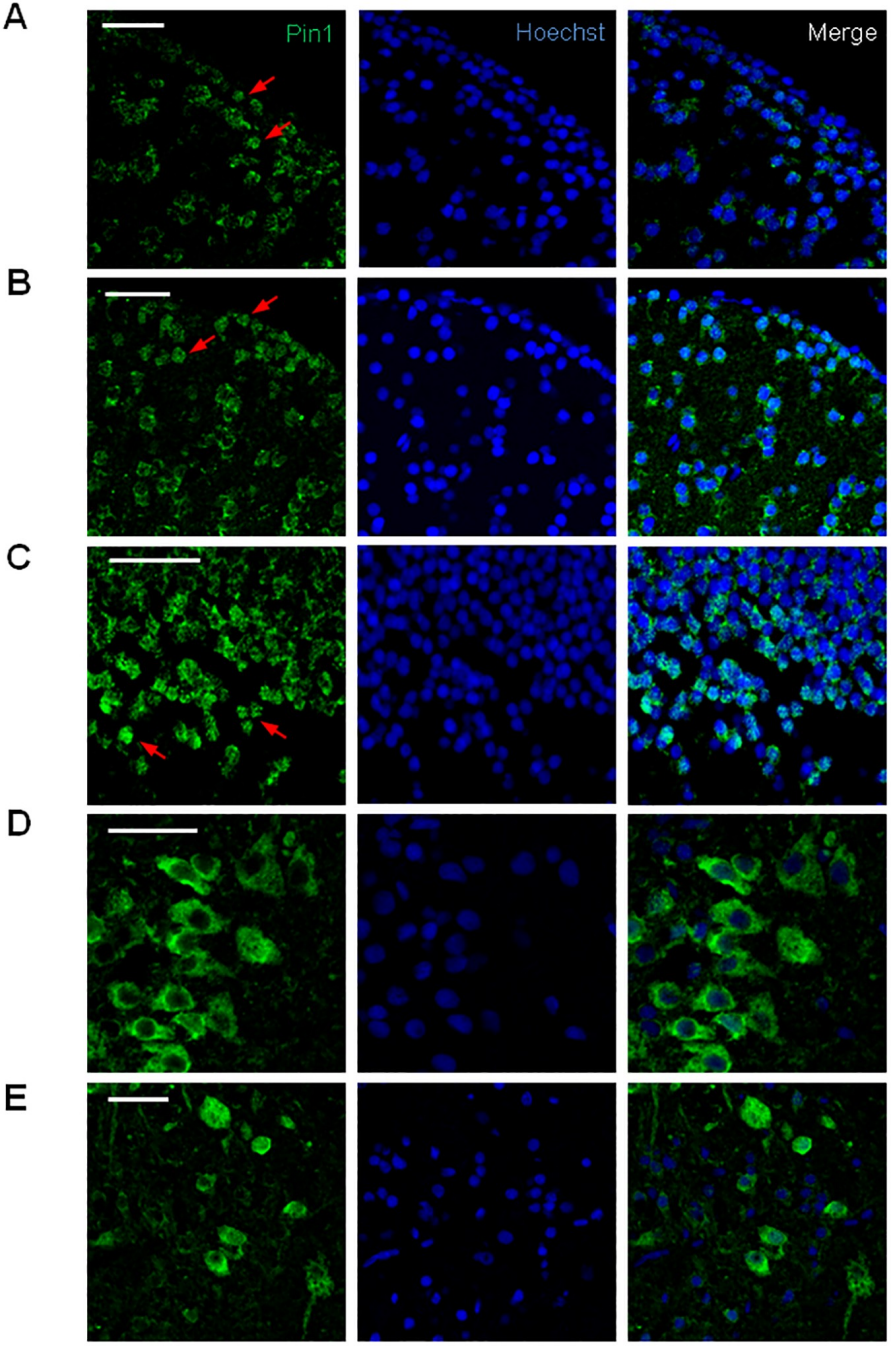
Pin1 subcellular localization in the adult zebrafish brain. Confocal Immunofluorescence analysis on brain coronal sections using Pin1 (green) as primary antibody. Nuclei were stained with Hoechst (blue). Digital magnification of selected areas from images shown in Fig 5 and S6 Fig. (A) lateral zone of dorsal telencephalic area, (B) diffuse nucleus of the inferior lobe, (C) periventricular gray zone of the optic tectum, (D) lateral zone of rostroventral medulla oblongata, (E) central area of caudal medulla oblongata. Scale bar = 25 μm.

We observed a considerable overlap between areas enriched in Pin1 and HuC/D expressing cells, which was particularly evident in the periventricular regions of the telencephalon and diencephalon and in the optic tectum. In accordance with the results obtained in embryos, we found numerous cells showing a cytoplasmic concentration of Pin1 signal. Strikingly, complete absence of co-localization between Pin1 signal and nuclear staining was observed in some cells, strongly suggesting a marked nuclear exclusion (Fig 7). However, we also found cells with a signal distribution suggestive of nuclear accumulation as well as cells with no evident differential subcellular localization (Fig 7, red arrows). In summary, our results show that Pin1 is present in particular cell types in the adult brain and that different subcellular localizations exist.

These findings prompted us to analyze if a cytoplasmic accumulation of Pin1 also exists in mammals. Cross sections were obtained from adult mouse brains and analyzed by immunofluorescence with the Pin1 antibody (S7 Fig). Several areas with abundant Pin1 expression were found, including the dentate gyrus of the hippocampus, the cerebral cortex and the cerebellar intermediate layer. In agreement with the idea that Pin1 expression is differentially regulated in specific cell types, we also found regions with Pin1 negative cells. In addition, a large number of cells with cytoplasmic accumulation of Pin1 was observed, including cells with nuclear exclusion of Pin1 signal, supporting our results on zebrafish and strongly suggesting that in several cell types higher cytoplasmic Pin1 levels are required *in vivo*.

## Discussion

Despite several studies analyzing the effects of Pin1 on protein substrates or specific signaling pathways, a more comprehensive understanding of its biological role has been elusive. In this regard, an important question is how Pin1 function is regulated *in vivo* to cause different and even opposing effects on cell behavior under specific circumstances. The dependence on the particular combination of protein substrates present in a specific cell type and on the activation of signaling pathways able to phosphorylate Pin1 binding sites have complicated the rationalization of its function. Moreover, the presence of a phosphate group on Ser or Thr hampers the ability of other prolyl isomerases to act on S/T-P sites. Therefore, Pin1 is the only enzyme able to link phosphorylation signaling with peptidyl-prolyl isomerization. In addition, Pin1 may impact on the action of phosphatases, since several of them are specific for *cis* or *trans* conformations. These unique characteristics underline the intimate interrelation between Pin1 and the phosphorylation landscape. Considering this complex scenario, studies in animal models may provide novel insights on the role of Pin1, useful to understand proline-directed phosphorylation signaling and the consequences of Pin1 deregulation. In this work we have characterized the expression of Pin1 during development in zebrafish. Our results revealed that Pin1 expression is highly regulated during embryogenesis. Moreover, we found that abnormally high Pin1 levels may affect specific regions of the embryo, further supporting the existence of precise regulatory mechanisms.

Perhaps due to the fact that Pin1 knock-out mice and flies are viable, little attention was put on the regulation of Pin1 expression during embryogenesis. The presence of Pin1 was reported in Primordial Germ Cells from E7.5 to E13.5 in mouse embryos [54]. Pin1 was also found in the brain cortex of mouse embryos after E12.5 [53]. In zebrafish, Pin1 protein was detected by western blot on total lysates of 24 hpf embryos [48]. Nevertheless, studies on Pin1 distribution in other embryonic tissues, as well as a more comprehensive characterization of changes in gene expression during development, have not been reported. We found that zebrafish *pin1* mRNA is maternally inherited, but mRNA levels are markedly reduced after 4 hpf, indicating that different regulatory mechanisms are activated to control *pin1* expression on later stages. Even if not conclusive, these observations suggest that the presence of Pin1 is required at the initial stages of development, before *de novo* transcription starts. Basing on the absence of evident morphological abnormalities during development of Pin1 knock-out mice and flies, it was suggested that it is dispensable for embryogenesis. Accordingly, Pin1 knock-down in zebrafish embryos by morpholino injection did not result in any evident morphological phenotype up to 24 hpf [48,50], and only a mild increase in apoptotic cells was observed. However, Pin1 may be crucial for successful development under stress conditions, as suggested by evidences showing that Pin1 knock-down reduced apoptosis in response to ionizing radiation in zebrafish embryos [48]. Therefore, embryos lacking Pin1 may fail to activate an efficient DNA damage response, retaining cells with genomic alterations.

Furthermore, our work suggests that Pin1 may play a role in specific regions of the embryo since its expression is precisely regulated during the analyzed stages to achieve different mRNA and protein distribution. We found a change from a non-restricted Pin1 mRNA pattern to a markedly restricted one, showing a notable concentration in the embryo head after 24 hpf. In contrast, mRNA levels were reported to be similar in several tissues from adult mice [38]. The observed distribution, displaying a higher accumulation of *pin1* mRNA in the cerebellum, dorsal telencephalon and diencephalon is suggestive of a specific role for Pin1 in the developing brain. These results are in agreement with recent reports showing that Pin1 is important for the development of the nervous system. Indeed, reduced number of upper layer cortical neurons was described in neonatal Pin1 knock-out mice [53], while altered axon guidance was found in cranial nerves and entorhino-hippocampal projections from Pin1 knock-out mouse embryos. Specifically, in 24 hpf zebrafish embryos, Pin1 knock-down alleviated the defects in motor neuron growth caused by impaired Sema3 signaling [50].

Our results indicate that *pin1* expression is regulated at multiple levels, since the protein is present at comparable levels both in the head and trunk of the embryos, despite a highly polarized pattern of mRNA distribution. Different mRNA stability, processing and/or translation rates may be required in different regions of the embryo. In particular, higher Pin1 mRNA steady-state levels in the head may be necessary to ensure fast and efficient translation, allowing rapid protein turnover in response to changing conditions or stress situations. This may help to eliminate proteins with complex post-translational modification codes or to create transient changes in protein concentration in microdomains. In addition, our findings showed cytoplasmic concentration in specific stages of development and in some cells in the adult brain strongly suggesting that Pin1 subcellular localization is tightly regulated.

Furthermore, we show for the first time that some cells display a dramatic reduction of nuclear Pin1 *in vivo*. Taking into consideration the variety of cytoplasmic and nuclear Pin1 targets, the possibility to re-localize Pin1 according to different cell contexts may represent an efficient mechanism to regulate its activity. Nuclear concentration of Pin1 was observed in several cell lines, however, studies in primary cultures or tissues have shown that Pin1 may be abundant in the cytoplasm as well. Specifically in neurons, the subcellular localization of Pin1 has been debated, with reports from *postmortem* human brains of both nuclear concentration and exclusive cytoplasmic localization [55–57]. However, recent evidences from immunofluorescence studies showed Pin1 cytoplasmic localization in primary cultures of mouse cortical neurons [58] and in axons from cultured dorsal root ganglia neurons [50]. Also, cell fractionation in human brains [59] and cultured embryo Neural Progenitor Cells [53] showed that Pin1 may be present in both compartments at comparable levels without evident nuclear concentration. Our work contributes to shed light on this issue showing that cells with fairly different Pin1 localization exist *in vivo*, and suggesting that subcellular distribution may be dynamically regulated according to cell type and context.

Post-translational modifications are likely to play a key role in the regulation of subcellular localization, as suggested by previous evidences from cell lines [7,8,43–45]. Moreover, considering that Pin1 protein levels do not seem to radically change among different regions of the zebrafish embryo or different human or mouse tissues [29,38], post-translational modifications may be crucial to achieve differential regulation of Pin1 function. We showed that the relative abundance of Pin1 phosphorylated isovariants was clearly different between 4:30 hpf and 24 hpf, supporting the notion that changes in these post-translational modifications during development may contribute to regulate subcellular localization. It is interesting to note that despite the high similarity between zebrafish and human Pin1 primary sequence the isoelectric points of the isovariants are drastically different, since in the case of the human protein, isoelectric points ranging from 6.0 to 8.9 were reported, the latter corresponding to the unmodified polypeptide [60]. Among the kinases that phosphorylate Pin1, DAPK1, PKA and Aurora A were proposed to inhibit nuclear localization and are therefore potentially responsible for a similar localization *in vivo* [7,43,44]. Accordingly, a nuclear localization signal between aminoacids 45 and 85 was reported [61], which harbors at least three residues that were shown to be post-translationally modified.

Our results from mRNA injection experiments give further support to the idea that precise regulation of Pin1 is necessary for normal embryogenesis, since alteration of facial structures in 3 dpf embryos was observed upon Pin1 overexpression. Pathological effects are often ascribed to elevated Pin1 levels. However, overexpression studies in vertebrates were limited to mammary epithelium [62], postnatal neurons [26] or the first rounds of division in *X*. *laevis* embryos [63]. Our results contribute to further characterize the consequences of Pin1 overexpression in animal models and suggest that some cell types may be particularly sensible to abnormally high Pin1 levels, since we did not observe evident morphological alterations in other regions of the embryo. Moreover, we showed that Pin1 mRNA microinjection enhanced p53-dependent apoptosis in 24 hpf embryos. Previous evidences from a mouse model of Huntington’s disease showed that induction of p53 targets p21WAF1 and PUMA is reduced in Pin1 knock out mice [27]. Our findings further support the role of p53 as a relevant Pin1 substrate in vivo and identify a specific stage of development where Pin1 overexpression affects cell physiology through activation of a p53-dependent response. In contrast to the pro-neoplastic effect of Pin1 overexpression in mammary tissue of adult mice [62], the increment in Pin1 levels in zebrafish embryos induced apoptosis and did not triggered an hyperproliferative response. Along with evidences from other authors, our results support the notion that, depending on the cell type and context, Pin1 high levels may promote pro-apoptotic or pro-oncogenic responses.

A differential regulation of Pin1 function is likely to be operated also in the adult brain, as suggested by the presence of a specific expression pattern and cells with different subcellular localization. In adult zebrafish brains Pin1 was restricted to particular areas, in contrast with the abundant expression observed in embryos. Accordingly, reduced Pin1 expression was reported in the brain cortex of neonatal mice, comparing with E15.5 embryos [53], further suggesting that during development high Pin1 levels may be beneficial. In adult zebrafish brains we found Pin1 in regions that show proliferative activity [64], such as telencephalic and diencephalic periventricular zones, as well as the olfactory bulbs. These observations pose the question whether Pin1 is involved in adult neurogenesis. Pin1 was proposed to enhance differentiation of Neural Progenitor Cells in mouse embryo cerebral cortex [53]. Conversely, Pin1 was shown to cooperate with the maintenance of self-renewal and pluripotency in Embryonic Stem Cells and Induced Pluripotent Stem Cells [65]. Therefore, Pin1 may be involved in different aspects of stem cell behavior, depending on the array of phosphorylated substrates present during self-renewal or differentiation. Neurogenic niches in the adult zebrafish brain contain different cell types including stem cells, transit amplifying precursors, committed progenitors and neurons. Pin1 was found in embryonic neurospheres and was suggested to be present in Nestin positive cells in the developing cortex of mouse embryos [53]. However, the presence of Pin1 in adult Neural Stem Cells has not been studied yet. Our results confirmed that Pin1 expression regions overlap with areas containing neurons, as judged by HuC/D staining. Future studies to characterize the cell types that express Pin1 in neurogenic niches will be important to understand the role of Pin1 in adult neurogenesis.

Collectively, our work revealed that zebrafish Pin1 is subjected to a complex regulation in the developing embryo and in the adult brain. In the last decade, zebrafish has been validated as a powerful model to study human diseases [66]. Noteworthy, the ability to regenerate injured brain makes zebrafish an interesting model to study adult neurogenesis [67]. Taking into consideration the emerging role of Pin1 as a critical factor in neurodegenerative diseases and cancer, our findings provide novel clues that may be helpful to understand the underlying mechanisms and to develop novel disease models.

## Materials and methods

### Fish

Adult zebrafish (Danio rerio, strains AB and *tp53*^*zdf1/zdf1*^) were maintained at 28°C on a 14 hours light/10 hours dark cycle as previously described [68]. All embryos were staged according to development in hpf or dpf at 28°C [69], as well as handled in compliance with relevant national and international guidelines. Protocols were approved by the Commission of Bioethics for Research, Facultad de Ciencias Bioquímicas y Farmacéuticas—Universidad Nacional de Rosario, which has been accepted by the Ministerio de Salud, Argentina http://www.saludinvestiga.org.ar/comites.asp?num_prov=13).

### RNA extraction and RT-PCR analysis

Total RNA from embryos at different developmental stages was obtained using TRIZOL Reagent (Invitrogen) following the manufacturer’s instructions. The integrity of RNA was checked using agarose gel electrophoresis. Purified RNA was incubated with RQ1 DNAse (Promega) and retro-transcribed with SuperScript II enzyme (Invitrogen) using oligodT according to manufacturer instructions. Real time PCR was performed in an Eppendorf Realplex2 thermocycler using SYBR green master mix (Invitrogen). After an initial denaturation step (94°C for five minutes), 40 amplification cycles were performed, consisting of a denaturing step of 20 seconds at 94°C, an annealing step of 30 seconds at 63°C and an extension step of 30 seconds at 68°C, and a final extension step of 10 minutes (min) at 68° C. ef1α an d rpl13a were used as endogenous controls for gene expression normalization. The following primers were used for qPCR: EF1αq fw 5’-CTGGAGGCCAGCTCAAACA T-3’, EF1αq rv 5’-ATCAAGAAGAGTAGTACCGCTAGCATTAC-3’, RPL13Aq fw 5’-TCTGGAGGACTGTAAGAGGTATGC-3’, RPL13Aq rv 5’-AGACGCACAATCTTGA GAGCAG-3’, PIN1q fw 5’-GGCGTCTCAGTTCAGCGACT 3-’, PIN1q rv 5’-ACCGCT CATGTCTCCAACCT-3’. Relative gene expression values were calculated using qBASE [70]. For end point PCR the following primers were used: PIN1 fw 5’-AAAAGAATTCAATGTCCGACGACGAGAAGCTG-3’, PIN1 rv 5’-AAA ACTCGAGGGCTGGTTATCCGGTTCTCAAGA-3’, EF1α fw: 5’-CTGGAGGCCAGCT CAAACAT-3’, EF1αrv: 5’-ATCAAGAAGAGTAGTACCGCTAGCATTAC-3’.

### Expression constructs

In order to clone the zebrafish *pin1* coding sequence (NM_200748.1), the following primers were designed containing linkers for restriction enzyme digestion: PIN1FW: 5’–A AAAgaattcaATGTCCGACGACGAGAAGCTG-3’ and PIN1RV: 5’-AAAActcga gGGCTGGTTATCCGGTTCTCAAGA-3’. PCR was carried out using the cDNA obtained from total RNA of 1-cell stage embryos as a template. The amplified product cloned into pGEM-T-Easy (Promega) to generate pGPin1. The pT2-Pin1 plasmid was constructed by subcloning *pin1* coding sequence in the pT2AL200R150G vector [71]. The plasmid pGPin1 was used as a template for PCR, using the primers: PIN1T2FW 5’-AAAAAAGCTTATGTCCGACGACGAGAAGCTG-3’ and PIN1T2RV: 5’-AAAAGGA TCCGGCTGGTTATCCGGTTCTCAAGA-3’, which contain linkers for *Hind*III and *Bam*HI. Following the digestion of the PCR product and the plasmid, both were purified after agarose gel electrophoresis, using Qiaex II Gel Extraction Kit (QIAGEN) and ligated using T4 DNA ligase (Promega). The constructs pCMVSP6-EGFP-Pin1 and pCMVSP6-EGFP-WW were generated using Gateway technology (Invitrogen).Full length zebrafish Pin1 or WW domain coding sequence was subcloned into pME-EGFP to generate pME-EGFP-Pin1 or pME-EGFP-WW. Each plasmid was recombined using Gateway technology (Invitrogen) with p5E-CMV, p3E-polyA and PDestTol2CG2 [72] to generate pCMVSP6-EGFP-Pin1 or pCMVSP6-EGFP-WW, which contains the corresponding coding sequence fused to EGFP (EGFP-Pin1 or EGFP-WW), flanked by the SP6 promoter at the 5’ end and the SV40 polyadenylation signal at the 3’ end. In addition, the coding sequences were placed under the control of CMV promoter, to allow expression in eukaryotes. pCMVSP6-EGFP was made recombining pME-EGFP with a similar strategy. Site directed mutagenesis to introduce C109A mutation was performed by three PCR reactions using pCMVSP6-EGFP-Pin1 as a template. For the first PCR, the primer EGFPBamFw: 5’-AAAGGATCCATGGTGAGCAAGGGCGAGG-3’was used in combination with the primer Pin1C109ARv: 5’-CCTCGCTGAGCTGGCGTCGCTGAACTGAG-3’. For the second, the primer Pin1C109AFw: 5’-CTCAGTTCAGCGACGCCAGCTCAGCGAG-3’ was used in combination with Pin1XbaRv: 5’-AAATCTAGATTATCCGGTTCTCAAGATGATGTG-3’. The two PCR products generated overlap at the site of mutagenesis. For the third PCR, the two respective PCR products were annealed, a fill-in reaction was performed, and the resulting DNA fragment was amplified using EGFPBamFw and Pin1XbaRv primers. The final product was inserted into pCS2+ and verified by sequencing.

### Whole-mount mRNA in situ hybridization

Embryos were staged and fixed overnight in 4% (w/v) paraformaldehyde (PFA) in phosphate saline buffer (PBS) at 4°C. After washing, embryos were stored in methanol at -20°C until used. For 48 hpf embryos melanogenesis was inhibited by incubation in 0.0045% (w/v) 1-Phenyl-2-Thiourea after gastrulation. The Pin1 antisense probe containing the complete coding region was synthesized using pGPin1 linearized with *Spe*I as a template. For control sense probe, pGPin1 linearized with *Sac*II was used. DNA was run on agarose gel and purified using Qiaex II Gel Extraction Kit. Digoxigenin-UTP-labeled riboprobes were prepared using DIGRNA labeling kit according to the manufacturer’s instructions (Roche Diagnostics, Mannheim, Germany). Probes were purified by ethanol precipitation. The procedure for whole-mount in situ hybridization was carried out as previously described [73]. Briefly, after rehydration embryos were permeabilized with proteinase K solution, washed, prehybridized and incubated overnight with the RNA probes. After extensive washing, embryos were blocked and incubated with anti-digoxigenin (Roche). Following washing in PBS with 0.1% (v/v) Tween-20 (PBST), signal was developed in NBT/BCIP solution (Roche).

### Embryonic extract preparation and western blot analysis

Zebrafish embryos (~100 embryos from different stages) were dechorionated in eppendorf tubes either with pronase (50 μg/ml) and swirl for 10–15 min or manually with forceps in E3 medium (5 mM NaCl, 0.17 mM KCl, 0.33 mM CaCl_2_, and 0.33 mM MgSO_4_, pH 6.8–6.9). After washing with E3, the embryos were resuspended in Ginzburg media (55 mM NaCl; 1.8mM KCl; 1.25mM NaHCO_3_; 27 mM CaCl_2_), and deyolked by passing through a 200 μl tip, and shaking for 5 min at 200 rpm. Then they were centrifuged for 30 seconds at 600 rpm and the supernatant discarded. Next, the embryos were rinsed with Washing buffer (110 mM NaCl; 3.5 mM KCl; 2.7 mM CaCl_2;_ 10 mM Tris-HCl (pH 8.5)) and shaked for 2 min at 200 rpm. Finally, the embryos were centrifuged for 30 seconds at 600 rpm and the supernatant discarded. Deyolked embryos were homogenized in two volumes of ice-cold extract buffer (20 mM HEPES pH 7.6; 150 mM NaCl; 10 mM MgCl_2_; 1 mM PMSF; 1 mM DTT; 1 mM EDTA; 0.5% (v/v) Triton X-100; Protease Inhibitor Cocktail (P8340, Sigma); 5mM NaF and 1mM Na_3_VO_4_) in Potter-Elvehjem tissue grinder on ice. Homogenates were centrifuged for 30 min at 14000 rpm at 4°C. Supernatants were diluted in Sample Buffer 5x (250 mM Tris-HCl (pH 7.5); 8% (w/v) SDS; 20% (v/v) Glycerol; 0.4 M DTT; and 0.1% (w/v) Bromophenol Blue) and incubated during 5 min at 95°C. Samples were centrifuged for 5 min at 14000 rpm and the samples were loaded onto a 15% gel for SDS–PAGE. The proteins were blotted to a PVDF membrane (Biorad) and stained for 10 min with Ponceau S red solution (0.1% (w/v) Ponceau S red in 1% (v/v) acetic acid) at room temperature. The membrane was washed several times with PBST until complete elimination of the staining. Then the membrane was incubated in blocking buffer (PBST supplemented with 5% (w/v) of skimmed milk) for 1 hour. After washing with PBST, the membrane was incubated with primary antibody diluted in blocking buffer. The membrane was washed with blocking buffer, and then incubated with anti-rabbit Ig HRP-linked antibody (Jackson) diluted 1/10000 in blocking buffer for 1 hour at room temperature. After washing with PBST and PBS, membranes were developed with SuperSignal West Pico Chemiluminescent kit (Thermo Scientific) using X-ray films (Amersham). Pin1 polyclonal antibody was obtained by immunization of rabbits with purified recombinant human Pin1 fused to GST obtained from bacteria. The antibody was affinity-purified using a GST-Pin1 resin. The eluted antibodies were passed through a GST-cross linked resin to eliminate anti GST immunoglobulins. The specificity of the antibody was tested by western blot in human cell lines silenced for Pin1 and in Mouse Embryo Fibroblasts from wt or knock out Pin1 mice [34]. The following commercial antibodies were used: γ-tubulin (T6557 Sigma); anti actin (A2066 Sigma), and anti GFP (Ab290 Abcam).

### Purification of recombinant proteins and pre-absorption assays

*Escherichia coli* BL21DE3 strains were used to express proteins from pGEX-2T or pGEX-Pin1 constructs [19,34]. One ml of overnight culture was used to inoculate 10 ml of LB containing 100 g/ml ampicillin. Expression was induced by addition of (IPTG) for 3 hours at 30°C. Cells were harvested by centrifugation and resuspended in 10 ml GST purification buffer (PBS, 5mM EDTA, NP40 1% (v/v)) with PMSF 1 mM, Protease Inhibitor Cocktail (Sigma), NaF 5mM, Na_3_VO_4_ 1mM and Lysozyme 0.05 mg/ml. Cells were disrupted by sonication and lysates were centrifuged at 15000 rpm for 5 min at 4°C. Supernatants were incubated with a suspension of 200 μl GSH-sepharose beads (GE Healthcare Life Sciences) in GST purification buffer for 2 hours at 4°C in rocking wheel. Subsequently, beads were washed three times with GST purification buffer at 4°C and three times with prechilled PBS at 4°C. Elution was achieved in GST elution buffer (0.1 M TrisHCl pH 8, 100 mM NaCl, 5% glycerol, 20 mM GSH) for 1 hour at 4°C followed by centrifugation at 2000 rpm for 2 min at 4°C. Finally, eluates were dialyzed against PBS overnight at 4°C. For the pre-absorption assays, 0.4 μg of rabbit Pin1 polyclonal antibody was pre-absorbed with 1 μg of GST or 1 μg GST-hPin1, in order to achieve a molar relation of 1:10. The incubation was performed for 6 hours at room temperature before western blot or immunofluorescence experiments.

### Bidimensional denaturing gel electrophoresis

Extracts from 4:30 and 24 hpf embryos were precipitated to remove ionic species and other contaminants using 2-D CleanUp Kit (GE Healthcare) according to manufacturer´s instructions. The precipitates were resuspended in DeStreak Rehydration Solution plus 2% v/v IPG Buffer pH 3–10 NL (GE Healthcare) at 1 μg/μl of total protein for first dimension IEF. Isoelectric focusing was performed on Immobiline DryStrip pH 3−10 non-linear, 7 cm (GE Healthcare). The strips were hydrated overnight at 20°C with DeStreak rehydration solution containing 80 μg of total protein from each sample. Strips were then focused on a MultiphorTM II System (GE) using a PowerPack 3000 power supply (Bio-Rad). The temperature was set at 20°C and the voltage program was: 200 V x 20 min, 450 V x 20 min, 750 V x 20 min, 2000 V x 1 hour and 3000 V x 10 min. After isoelectric focusing, the strips were equilibrated in equilibration buffer (6 M urea, 75 mM Tris pH 8.8, 30% v/v glycerol, 2% w/v SDS, 0.006% w/v bromophenol blue) in two steps of 15 min, at 20°C and constant agitation. For the first step, DTT (10 mg/mL) was added and for the second, iodoacetamide (25 mg/mL) was included. For the second dimension, 15% SDS-polyacrylamide gels (15% w/v acrylamide, 390 mM Tris pH 8.8, 0.1% w/v SDS) were run. The strips were positioned alongside the molecular weight marker Precision Plus Protein Dual Xtra Standards (Bio-Rad). Following polyacrylamide gel electrophoresis separation, gels were transferred to nitrocellulose membranes (Amersham), and blots were blocked in 5% milk in PBST and probed with primary antibodies as described (Western blot section). Three independent samples were run for each stage. For lambda phosphatase treatment, 100 μg of zebrafish extracts prepared using modified extraction buffer (20 mM HEPES pH 7.6; 150 mM NaCl; 10 mM MgCl_2_; 1 mM PMSF; 1 mM DTT; 0.5% (v/v) Triton X-100 and Protease Inhibitor Cocktail (P8340, Sigma)) were incubated with 2500 U of Lambda protein phosphatase (New England BioLabs) for 2 h at 30°C.

### Immunofluorescence and confocal microscopy

Embryos were staged and fixed overnight in 4% PFA in PBS at 4°C. For whole mount immunofluorescence embryos were washed with PBS 3 times for 15 min each, and permeabilized with acetone 7 min at -20°C followed by 3 washes in PBS. For whole mount immunofluorescence embryos were blocked with 5% (w/v) bovine serum albumin (BSA) dissolved in PBSTX (0.1% Triton X-100 in PBS) for 1 hour at room temperature and then incubated overnight at 4°C with the primary antibodies diluted in 3% (w/v) BSA-PBSTX. The embryos were washed 3 times with PBS for 10 minutes each and incubated with the secondary antibodies (goat anti rabbit Alexa Fluor 488, Invitrogen, Cy3, Millipore) diluted 1:1000 in 3% (w/v) BSA-PBSTX for 1 hour at room temperature in the dark. Following 3 washes in PBSTX, nuclei were stained incubating the embryos with 0.1μg/ml Hoechst 333258 (Sigma) for 15 min. Finally, after 5 washes with PBS 5 min each, embryos were mounted in 1% (w/v) low melting agarose for confocal microscopy imaging. As primary antibodies polyclonal anti-Pin1 [19,34] or rabbit anti-phospho-Histone H3 (Ser10, Millipore) were used. For immunofluorescence on embryo sections fixed 48 hpf embryos were mounted in 1% agarose, dehydrated, cleared in xylene and embedded in paraffin. Sections of 5 μm were obtained from paraffin blocks, slides were deparaffinized in xylene, passed through graded alcohols, and rehydrated for staining. Brains were dissected from anesthetized adult fish (6 month to 1 year) washed in PBS and fixed in 4% PFA for 24 hours at 4°C. Samples were dehydrated and embedded in paraffin. Sections of 5 μm were obtained, deparaffinized and rehydrated for immunofluorescence. For immunofluorescence on paraffin sections, antigen unmasking was performed. To retrieve the Pin1 antigens, sections were incubated in citrate buffer (100 mM sodium citrate in PBS, pH 6.0) at 95°C for 30 min, whereas for HuC/D antigen retrieval, sections were pre-incubated in 1 M Tris-buffer (pH 8.0) at 100°C for 5 minutes and cooled down to room temperature over 15 minutes and washed three times for 5 minutes in PBS. The sections were then blocked for 1 hour at room temperature with 5% (w/v) BSA in PBSTX; they were then incubated overnight at 4°C with primary antibodies diluted in the blocking buffer. The primary antibodies were rabbit anti-Pin1 [34] and mouse anti-HuC/D (Invitrogen). Fluorescently coupled goat secondary antibodies (Alexa fluor 488; Invitrogen, Cy3, Millipore) were incubated at least for 4 hour at room temperature. Nuclear staining was performed with Hoechst 333258 (Sigma) for 15 min. Cultured cells were washed in PBS, fixed in 4% PFA for 20 min, and permeabilized in PBSTX. After washing, samples were blocked in 3% BSA in PBSTX and incubated with primary antibody diluted in blocking buffer overnight and the secondary antibodies were incubated 1 hour at room temperature. In all cases, a sample was processed in parallel omitting the primary antibody as a control. Confocal images were obtained using a Zeiss LSM 880 confocal microscope with a 20 x objective. Zen image acquisition software (Carl Zeiss) was used to analyze images.

### Transient transfections and cell culture

The mouse neuroblastoma cell line Neuro-2a was cultured in Modified Eagle's medium (MEM, Gibco) supplemented with 10% fetal bovine serum (FBS, Life Technologies), penicillin G (100 units/ml, Sigma) and streptomycin (100 μg/ml, Sigma). HEK-293 cells were maintained in Dulbecco’s modified Eagle’s medium (DMEM, Gibco) supplemented with 10% FBS, penicillin G/streptomycin. SH-SY5Y human neuroblastoma cells were grown in DMEM:F12 (Gibco) medium supplemented with 10% FBS and penicillin G/streptomycin. Cultured cells were maintained in a 5% CO_2_ humidified incubator at 37°C. To induce Neuro-2a or SH-SY5Y cell differentiation, the medium was changed to DMEM plus 2% FBS containing 10 μM all-*trans* retinoic acid for 24 h (Sigma). HEK-293 cells were transfected using Calcium Phosphate method. The day before transfection, 2.5x10^5^ cells were plated in 3.5 cm diameter dishes. For each sample a particle suspension of DNA-Ca_3_(PO_4_)_2_ was prepared as follows: 100 μl of 250 mM CaCl_2_ solution containing 1–2 μg of plasmid was added to 100 μl of 2X HBS (50 mM Hepes pH 7, 280 mM NaCl, 1.5 mM Na_2_HPO_4_). The mixture was incubated for 25–30 minutes at room temperature and added to the cells. After 18 hours incubation the medium was changed and the cells were further incubated for 24–48 hours.

### Morpholino and mRNA microinjection

Morpholino antisense oligonucleotide for Pin1: CTCTCTCTGCTCACTCTGGATGAG [48] and control morpholino synthesized by Gene Tools, were resuspended in sterile water at a concentration of 3 mM and delivered into one-cell stage zebrafish embryos by microinjection at a final concentration of 2 ng/μl in Danieau’s solution (58 mM NaCl, 0.7 mM KCl, 0.4 mM MgSO_4_, 0.6 mM Ca(NO_3_)_2_, 5.0 mM HEPES pH 7.6). MOs were injected into the yolk of embryos just below the cytoplasm. For mRNA synthesis, after plasmid linearization with *Not*I, the DNA was run in agarose gel and purified using Qiaex II Gel Extraction Kit and phenol-chloroform extraction followed by ethanol precipitation. Synthetic mRNAs were transcribed *in vitro* from linearized templates with the mMESSAGEmMACHINE kit (Ambion) using SP6 RNA polymerase according to manufacturer instructions. Embryos were obtained by natural mating and injected at the 1–2 cell stage using a gas-driven microinjection apparatus (MPPI-2 Pressure Injector, Applied Scientific Instrumentation; Eugene, OR). Embryos in E3 medium were injected with 5 nl of 100 ng/μl of EGFP-Pin1, EGFP, EGP-Pin1C109A or EGFP-WW mRNA diluted in Danieau’s solution and incubated at 28°C. Upon 5 hours, fluorescent embryos were selected and further incubated for morphological analysis. Embryos were anesthetized with 0.168 mg/ml tricaine (Sigma-Aldrich), mounted in 2% methylcellulose and examined with an Olympus DPT2 microscope equipped with an Olympus C-60 ZOOM digital camera.

### Apoptosis assay

Dechorionated embryos (24 hpf) were fixed in fresh 4% PFA in PBST overnight at 4°C, dehydrated using methanol (3 x 5 min), and stored overnight at 20°C. After gradual rehydration, the embryos were permeabilized with 5 μg/ml proteinase K for 10 min followed by 4% PFA fixation for 20 min. After several washes in PBST, embryos where incubated 2 hours at 37°C in a red fluorescent (TMR-red) TUNEL cell death detection reagent (In Situ Cell Death Detection Kit-TMR Red; Roche) and incubated 15 min in PBST containing Hoechst 333258 (Sigma) to counterstain nuclei. TUNEL positive cells in the head were scored considering as a limit a line parallel to the trunk drawn at the beginning of the otic vesicle.

## Supporting information

S1 Fig (A) Multiple Alignment analysis of Pin1 amino acid sequences from different species (COBALT). Conserved amino acids are highlighted with light grey boxes, absent residues are indicated by dashes. Dark grey boxes indicate residues that are relevant for substrate binding or catalytic activity (see text). *D*. *rerio*, NP_957042.1*; X*. *laevis*, NP_001089028.1; *M*. *musculus*, NP_075860.1*; R*. *norvegicus*, NP_001100171.1; *H*. *sapiens*, NP_006212.1. The percentage of identity between sequences from other species compared to *D*. *rerio* Pin1 is shown. (B) Schematic representation of human Pin1 showing identified phosphorylation and sumoylation sites.
(TIFF)

S2 Fig Pin1 polyclonal antibody specifically recognizes *D*. *rerio* Pin1. (A) Western blot analysis of extracts from HEK-293 cells transfected with pT2-Pin1 or pT2AL500R150G (pT2) probed with Pin1 antibody (upper panel) or GFP antibody (lower panel), (-) untransfected cells, (1) and (2) indicate two independent transfections. (B) Western blot analysis of embryonic extracts from the indicated stages with Pin1 antibody pre-absorbed with recombinant GST or GST-hPin1. (C) Whole-mount immunofluorescence of 4:30 hpf embryos with untreated Pin1 antibody (upper panels), Pin1 antibody pre-absorbed with recombinant GST (middle panels) or pre-absorbed with GST-hPin1 (lower panels). The insets show digital magnifications of selected regions from each image. (D) Zebrafish embryos were microinjected at 1 cell-stage with 1.5 or 6 ng of control or Pin1 specific morpholinos (MO), and upon 24 hours, western blot was performed on protein extracts using anti Pin1 and anti Actin as loading control (E) Whole-mount immunofluorescence of 6 ng Pin1 MO or control MO microinjected embryos at 24 hpf using Pin1 antibody, showing part of the head (upper panels), or trunk (lower panels). The insets show digital magnifications of selected regions from each image.
(TIFF)

S3 Fig Analysis of Pin1 expression in 48 hpf zebrafish embryo sections. Immunofluorescence was performed on 5 μm coronal sections from 48 hpf embryos that were fixed and embedded in paraffin. Pin1 polyclonal antibody (green) was used and nuclei were stained with Hoechst (blue). (A) horizontal section showing part of the midbrain and hindbrain, (B) coronal section of the ventral telencephalon, (C) coronal section showing part of the eye cup and of the lateral region of the diencephalon. OT: optic tectum, Cb: cerebellum, E: eye. Scale bar = 50 μm.
(TIFF)

S4 Fig (A) Confocal Immunofluorescence analysis of cultured Neuro-2a and SH-SY5Y cells using anti-Pin1 as primary antibody (green). Nuclei were stained with Hoechst (blue). Cells were plated and 24 hours later all-*trans* retinoic acid (RA, 10 μM) was added. Control cells were incubated in culture medium. Scale bar = 25 μm. (B) HEK-293 cells were transfected with pCMVSP6-EGFP, pCMVSP6-EGFP-Pin1, pCMVSP6-EGFP-WW and pCMVSP6-EGFP-Pin1C109A plasmids and upon 24 hours, western blot was performed on protein extracts using GFP antibody (left panel) or Pin1 antibody (right panel).
(TIFF)

S5 Fig Analysis of Pin1 expression in the adult zebrafish brain. Confocal Immunofluorescence analysis on brain coronal sections using Pin1 (green, upper panels) as primary antibody. Nuclei were stained with Hoechst (blue). (A) olfactory bulb (B) telencephalic lobe, (C) ventral diencephalon (D) midbrain, (E) cerebellum and medulla oblongata, (F) medulla oblongata (caudal) (G) medulla spinalis. Scale bar = 100 μm.
(TIFF)

S6 Fig Regions enriched in Pin1 expressing cells in the adult zebrafish brain. Confocal Immunofluorescence analysis on brain coronal sections using Pin1 (green) or HuC/D (red) as primary antibodies. Nuclei were stained with Hoechst (blue). (A) diencephalic ventricle (B) lateral zone of rostroventral medulla oblongata, (C) and (D) central area of caudal medulla oblongata, (E) lobus vagus, (F) lobus facialis. Cp: central posterior thalamic nucleus, LVII: lobus facialis, LX: lobus vagus, TPp: periventricular nucleus of posterior tuberculum. Scale bar = 50 μm.
(TIFF)

S7 Fig Analysis of Pin1 expression in the adult mouse brain. Confocal Immunofluorescence analysis on mouse brain coronal sections using Pin1 (green) as primary antibody. Nuclei were stained with Hoechst (blue). (A) cerebellum, (B) and (C) cortex, (D) dentate gyrus. Scale bar = 50 μm.
(TIFF)
