# Supplementary figures and images for: Dynamic regulation of Pin1 expression and function during zebrafish development
Source: PLoS One. 2017 Apr 20;12(4):e0175939. doi: 10.1371/journal.pone.0175939 (PMC5398671; doi:10.1371/journal.pone.0175939)

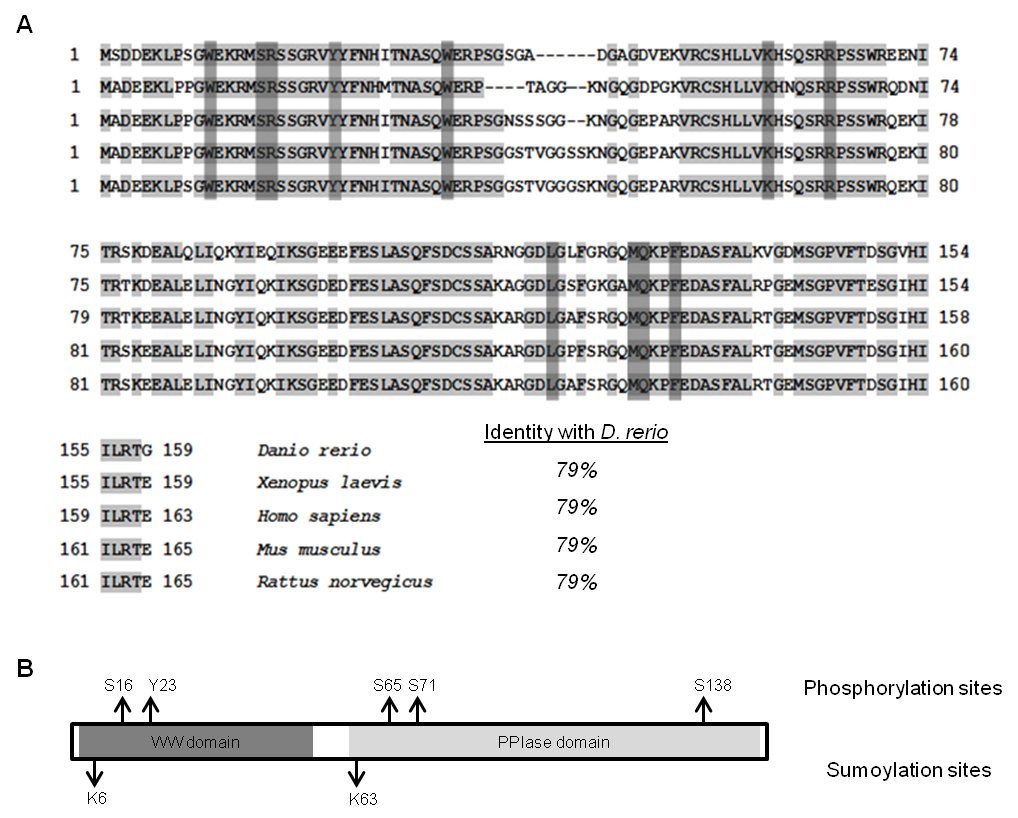

Supplement: S1 Fig — (A) Multiple Alignment analysis of Pin1 amino acid sequences from different species (COBALT). Conserved amino acids are highlighted with light grey boxes, absent residues are indicated by dashes. Dark grey boxes indicate residues that are relevant for substrate binding or catalytic activity (see text). D. rerio, NP_957042.1; X. laevis, NP_001089028.1; M. musculus, NP_075860.1; R. norvegicus, NP_001100171.1; H. sapiens, NP_006212.1. The percentage of identity between sequences from other species compared to D. rerio Pin1 is shown. (B) Schematic representation of human Pin1 showing identified phosphorylation and sumoylation sites. (TIFF) [file pone.0175939.s001.tiff]

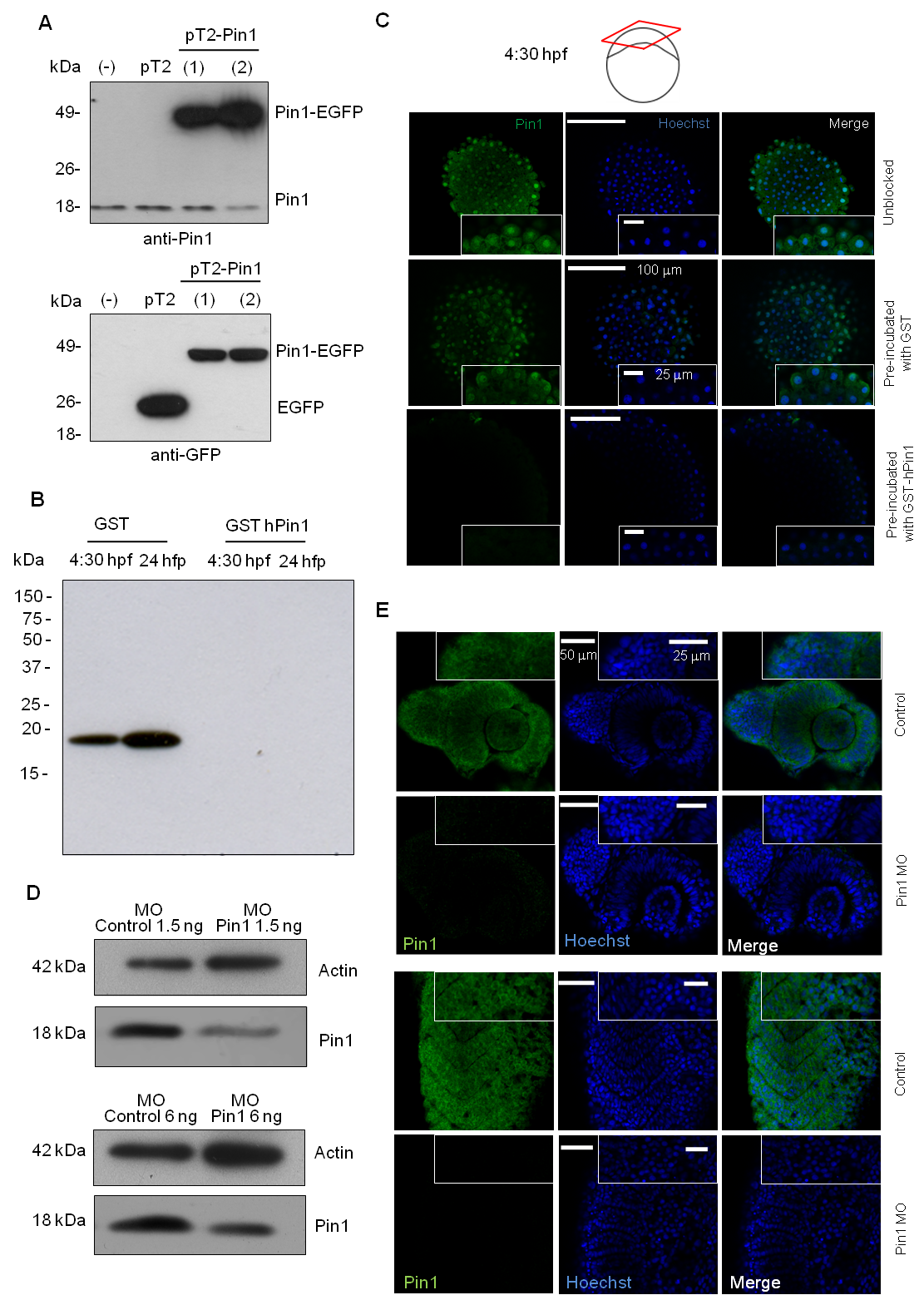

Supplement: S2 Fig — (A) Western blot analysis of extracts from HEK-293 cells transfected with pT2-Pin1 or pT2AL500R150G (pT2) probed with Pin1 antibody (upper panel) or GFP antibody (lower panel), (-) untransfected cells, (1) and (2) indicate two independent transfections. (B) Western blot analysis of embryonic extracts from the indicated stages with Pin1 antibody pre-absorbed with recombinant GST or GST-hPin1. (C) Whole-mount immunofluorescence of 4:30 hpf embryos with untreated Pin1 antibody (upper panels), Pin1 antibody pre-absorbed with recombinant GST (middle panels) or pre-absorbed with GST-hPin1 (lower panels). The insets show digital magnifications of selected regions from each image. (D) Zebrafish embryos were microinjected at 1 cell-stage with 1.5 or 6 ng of control or Pin1 specific morpholinos (MO), and upon 24 hours, western blot was performed on protein extracts using anti Pin1 and anti Actin as loading control (E) Whole-mount immunofluorescence of 6 ng Pin1 MO or control MO microinjected embryos at 24 hpf using Pin1 antibody, showing part of the head (upper panels), or trunk (lower panels). The insets show digital magnifications of selected regions from each image. (TIFF) [file pone.0175939.s002.tiff]

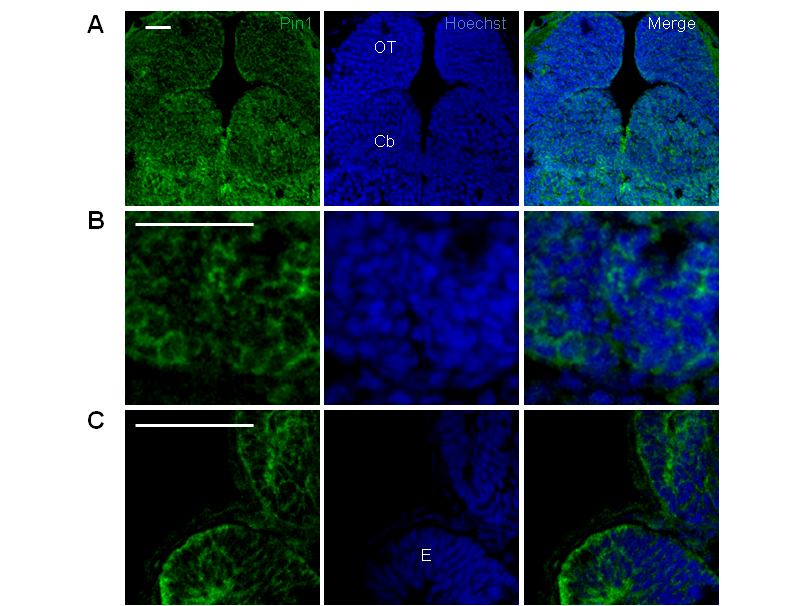

Supplement: S3 Fig — Immunofluorescence was performed on 5 μm coronal sections from 48 hpf embryos that were fixed and embedded in paraffin. Pin1 polyclonal antibody (green) was used and nuclei were stained with Hoechst (blue). (A) horizontal section showing part of the midbrain and hindbrain, (B) coronal section of the ventral telencephalon, (C) coronal section showing part of the eye cup and of the lateral region of the diencephalon. OT: optic tectum, Cb: cerebellum, E: eye. Scale bar = 50 μm. (TIFF) [file pone.0175939.s003.tiff]

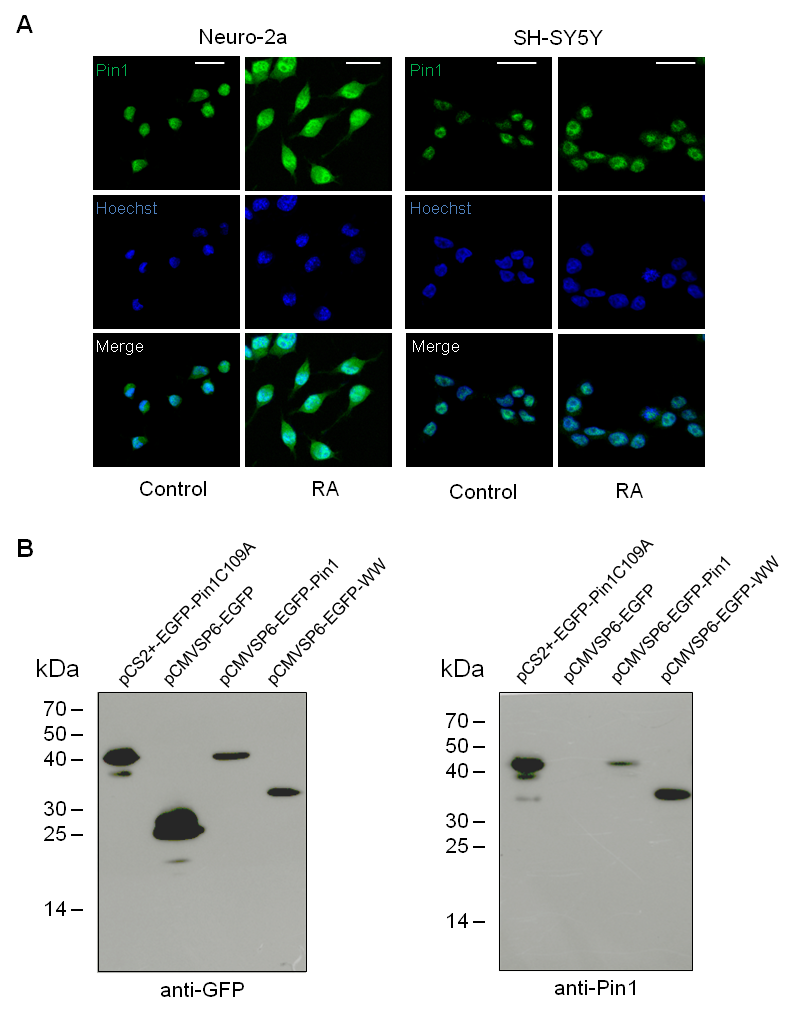

Supplement: S4 Fig — (A) Confocal Immunofluorescence analysis of cultured Neuro-2a and SH-SY5Y cells using anti-Pin1 as primary antibody (green). Nuclei were stained with Hoechst (blue). Cells were plated and 24 hours later all-trans retinoic acid (RA, 10 μM) was added. Control cells were incubated in culture medium. Scale bar = 25 μm. (B) HEK-293 cells were transfected with pCMVSP6-EGFP, pCMVSP6-EGFP-Pin1, pCMVSP6-EGFP-WW and pCMVSP6-EGFP-Pin1C109A plasmids and upon 24 hours, western blot was performed on protein extracts using GFP antibody (left panel) or Pin1 antibody (right panel). (TIFF) [file pone.0175939.s004.tiff]

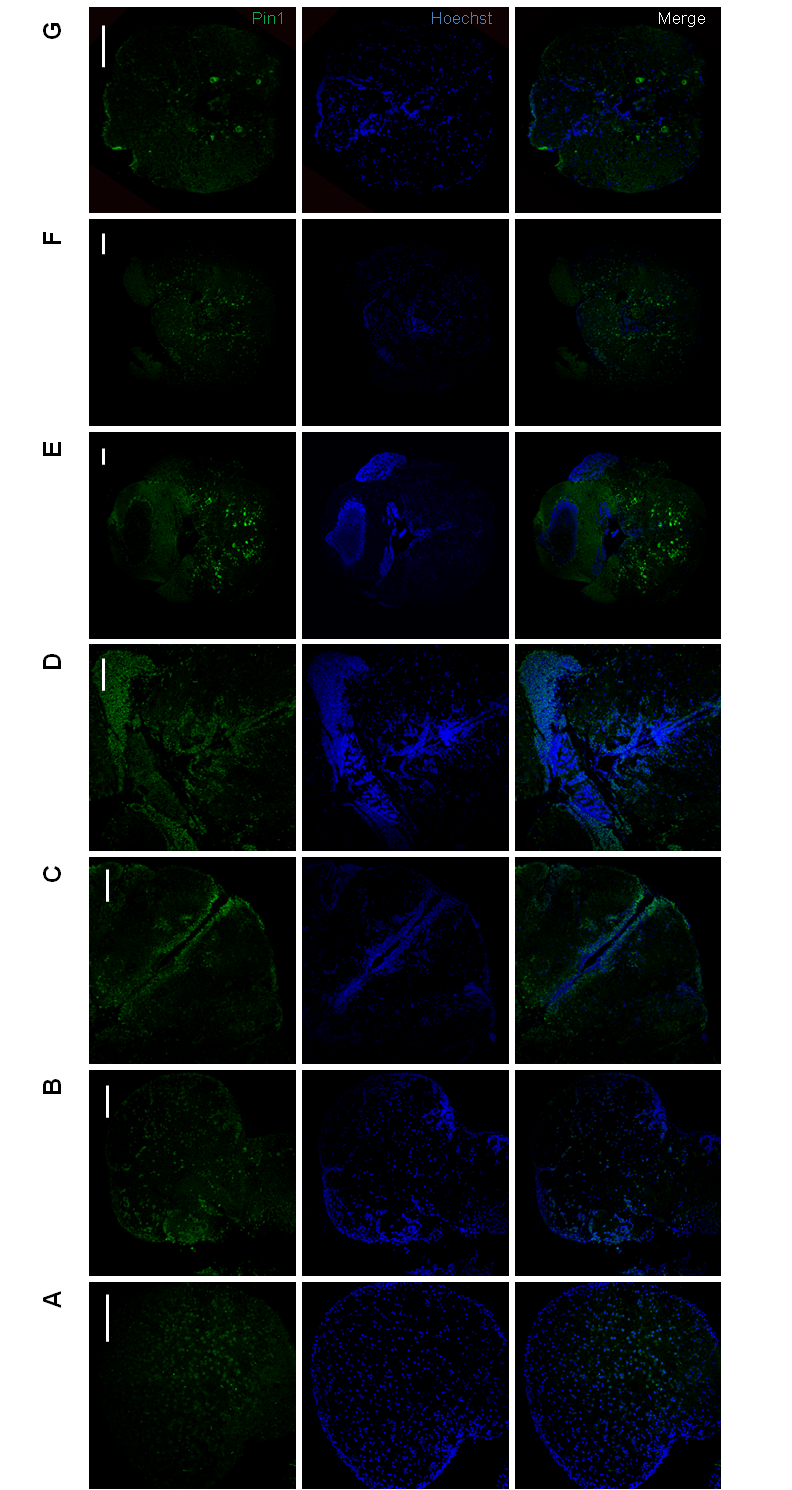

Supplement: S5 Fig — Confocal Immunofluorescence analysis on brain coronal sections using Pin1 (green, upper panels) as primary antibody. Nuclei were stained with Hoechst (blue). (A) olfactory bulb (B) telencephalic lobe, (C) ventral diencephalon (D) midbrain, (E) cerebellum and medulla oblongata, (F) medulla oblongata (caudal) (G) medulla spinalis. Scale bar = 100 μm. (TIFF) [file pone.0175939.s005.tiff]

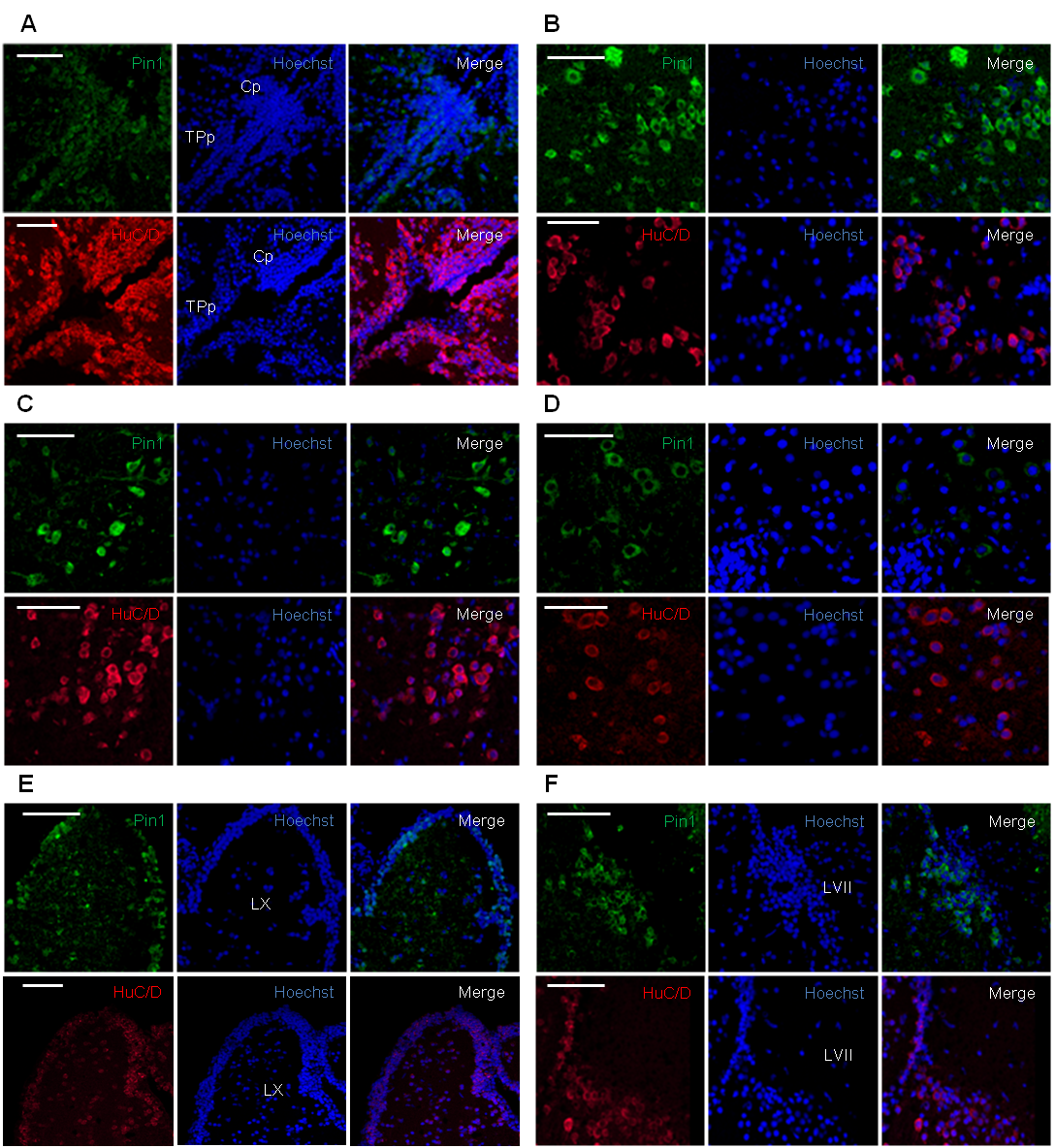

Supplement: S6 Fig — Confocal Immunofluorescence analysis on brain coronal sections using Pin1 (green) or HuC/D (red) as primary antibodies. Nuclei were stained with Hoechst (blue). (A) diencephalic ventricle (B) lateral zone of rostroventral medulla oblongata, (C) and (D) central area of caudal medulla oblongata, (E) lobus vagus, (F) lobus facialis. Cp: central posterior thalamic nucleus, LVII: lobus facialis, LX: lobus vagus, TPp: periventricular nucleus of posterior tuberculum. Scale bar = 50 μm. (TIFF) [file pone.0175939.s006.tiff]

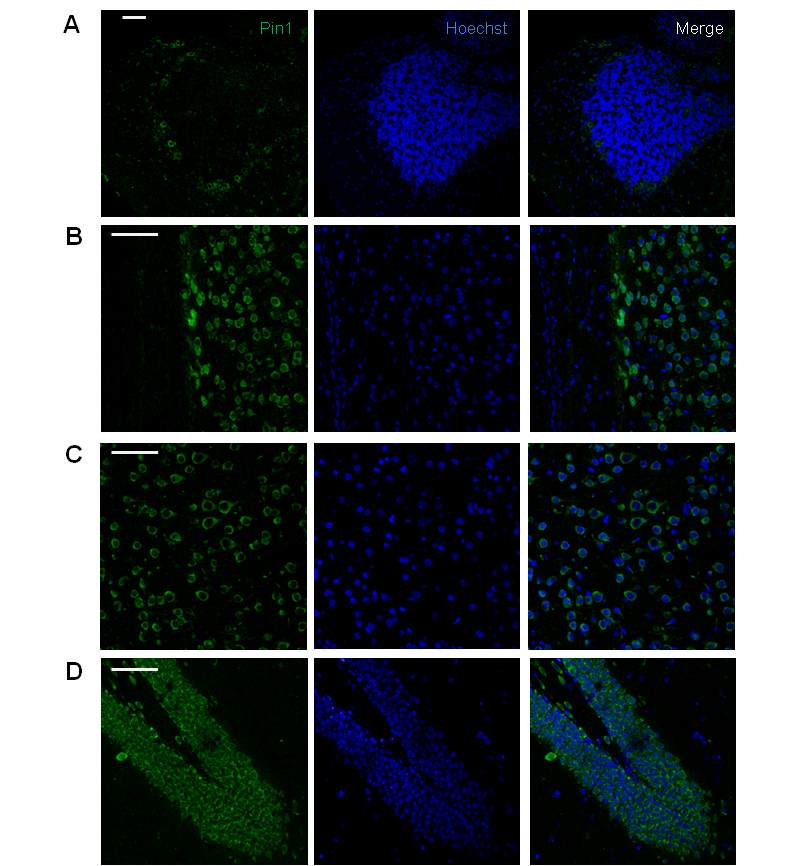

Supplement: S7 Fig — Confocal Immunofluorescence analysis on mouse brain coronal sections using Pin1 (green) as primary antibody. Nuclei were stained with Hoechst (blue). (A) cerebellum, (B) and (C) cortex, (D) dentate gyrus. Scale bar = 50 μm. (TIFF) [file pone.0175939.s007.tiff]
